# Exposure to Insecticides Modifies Gene Expression and DNA Methylation in Hematopoietic Tissues In Vitro

**DOI:** 10.3390/ijms24076259

**Published:** 2023-03-26

**Authors:** María del Pilar Navarrete-Meneses, Consuelo Salas-Labadía, María del Rocío Juárez-Velázquez, Dafné Moreno-Lorenzana, Fernando Gómez-Chávez, Alberto Olaya-Vargas, Patricia Pérez-Vera

**Affiliations:** 1 Laboratorio de Genética y Cáncer, Instituto Nacional de Pediatría, Mexico City 04530, Mexico; peachnavarrete@gmail.com (M.d.P.N.-M.); consusa@hotmail.com (C.S.-L.); rociojuarez@gmail.com (M.d.R.J.-V.); lorenzanadafne@gmail.com (D.M.-L.); 2 Maestría y Doctorado en Ciencia y Tecnología de Vacunas y Bioterapéuticos, Doctorado en Ciencias en Biotecnología, Laboratorio de Enfermedades Osteoarticulares e Inmunológicas, Instituto Politécnico Nacional-ENMyH, Mexico City 07738, Mexico; fgomezch@ipn.mx; 3 Unidad de Trasplante de Células Hematopoyeticas y Terapia Celular, Instituto Nacional de Pediatría, Mexico City 04530, Mexico; alberto.olaya@yahoo.com.mx

**Keywords:** permethrin, malathion, gene expression

## Abstract

The evidence supporting the biological plausibility of the association of permethrin and malathion with hematological cancer is limited and contradictory; thus, further studies are needed. This study aimed to investigate whether in vitro exposure to 0.1 μM permethrin and malathion at 0, 24, 48 and 72 h after cell culture initiation induced changes in the gene expression and DNA methylation in mononuclear cells from bone marrow and peripheral blood (BMMCs, PBMCs). Both pesticides induced several gene expression modifications in both tissues. Through gene ontology analysis, we found that permethrin deregulates ion channels in PBMCs and BMMCs and that malathion alters genes coding proteins with nucleic acid binding capacity, which was also observed in PBMCs exposed to permethrin. Additionally, we found that both insecticides deregulate genes coding proteins with chemotaxis functions, ion channels, and cytokines. Several genes deregulated in this study are potentially associated with cancer onset and development, and some of them have been reported to be deregulated in hematological cancer. We found that permethrin does not induce DNA hypermethylation but can induce hypomethylation, and that malathion generated both types of events. Our results suggest that these pesticides have the potential to modify gene expression through changes in promoter DNA methylation and potentially through other mechanisms that should be investigated.

## 1. Introduction

In recent decades, insecticide use has increased dramatically, as insecticides are employed in several human activities. Insecticides are widely used in agriculture, homes, aircraft, and hospitals. Thus, humans may potentially be exposed to these chemicals. Although multiple insecticides have been developed, pyrethroids and organophosphates are among the most commonly used [1,2,3,4,5]. The global use and exposure to pyrethroids and organophosphates are of concern because they have been associated with an increased risk of diverse diseases. Thus, the biological effects these pesticides exert on human health have attracted a great deal of interest [1].

The adverse effects insecticides can cause on humans have been associated with neurological alterations, inflammation, and cancer [3,4,6,7,8,9]. Different biological effects at the cellular and molecular levels have been reported as a consequence of pyrethroid and organophosphate exposures, including DNA damage, oxidative stress, epigenetic modifications, altered cell adhesion, endocrine disruption, apoptosis, altered proliferation/differentiation, and altered gene expression [10,11,12,13,14,15]. All these effects may underlie diseases associated with insecticide exposure, such as cancer. 

Exposure to pyrethroids and organophosphates can increase the risk of developing different types of cancer, particularly hematopoietic cancer. Epidemiological studies have suggested an association of pyrethroids with multiple myeloma in adults and acute lymphoblastic leukemia in children [16]. Similarly, exposure to organophosphates has been associated with an increased risk of lymphomas, leukemia, and multiple myeloma [17,18]. Although epidemiological studies have suggested an association of pyrethroids and organophosphates with hematological cancer, the experimental evidence showing if this is plausible is controversial [7,10,16,18,19,20]. Thus, it is necessary to further explore if pyrethroids and organophosphates can induce biological events at the cellular and molecular levels that could underlie or promote cancer development. 

Limited previous studies have assessed the effect of pesticides on gene expression and DNA methylation deregulation. In farmers exposed to mixtures of pesticides (dicamba, picloram, mesotrione, acetochlor, metolachlor, glyphosate, 2,4- dichlorophenoxyacetic acid (2,4-D), atrazine, aldrin, chlordane, DDT, dieldrin, heptachlor, lindane, toxaphene, and malathion), it was reported that DNA methylation changes occurred in whole blood. In this study, a total of 162 CpGs were differentially methylated in relation to 8 active ingredients (dicamba, picloram, mesotrione, aceto- chlor, metolachlor, glyphosate, atrazine, and malathion) [21]. In an additional study, it was reported that organophosphates induce changes in CpGs methylation in blood samples from controls and Parkinson’s disease patients. The most over-represented pathways were the nicotinic and muscarinic acetylcholine receptor signaling pathways [22]. These studies are particularly relevant and show that pesticides are capable of inducing DNA methylation modifications that could potentially modify gene expression. Unfortunately, these studies did not look for gene expression changes and included a questionary-based assessment of mixtures of pesticides, making it difficult to determine the effects of specific compounds. Regarding gene expression, for other pesticides such as paraquat, glyphosate and phosphonomethyl, changes in gene expression of DNA repair genes in farmers exposed to these chemicals have been reported [23]. However, in this study, permethrin and malathion were not included. An integrated bioinformatic study that included gene expression, microRNA expression, and DNA methylation dataset analysis reported that pesticides can modulate the expression levels of different genes and induce different epigenetic alterations in the expression levels of miRNAs and in the modulation of DNA methylation status. They found that two genes, *SLC44A1* and *FANCC*, were consistently deregulated in the different datasets [24]. Together, these studies show that pesticides can induce genetic and epigenetic changes that may be linked to several health issues. However, studies assessing the effect of individual pesticides are missing. To date, there are no studies evaluating the effect of permethrin and malathion on gene expression in vitro together with changes in DNA methylation.

In this study, we assessed whether in vitro exposure to permethrin, a very common pyrethroid, and malathion, a widely used organophosphate, can induce gene expression and DNA methylation changes in hematopoietic tissues. We analyzed mononuclear cells from bone marrow and peripheral blood (BMMCs and PBMCs) exposed in vitro to 0.1 μM of permethrin and malathion at 0, 24, and 48 h after culture initiation using RNA and DNA microarrays. 

## 2. Results

### 2.1. Changes in Gene Expression of BMMCs and PBMCs Exposed to Insecticides

**Figure 1:**
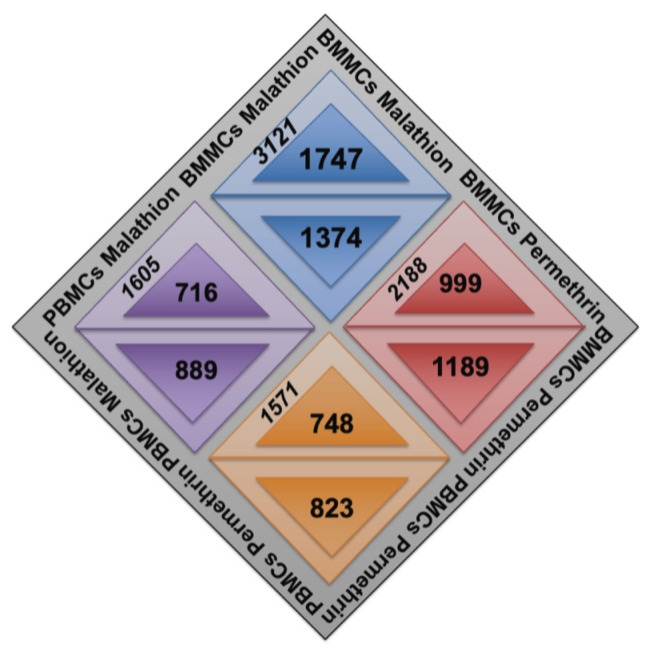
Distribution of genes with low expression (upside down triangles) and with overexpression (triangles looking up) on each microarray. Gene expression was analyzed in PBMCs and BMMCs exposed in vitro to permethrin or malathion. Each colored rhombus belongs to a specific treatment condition.

Permethrin and malathion induced changes in gene expression compared to cells exposed to DMSO (Figure 1). A higher number of genes (3121) with altered expression was detected in BMMCs exposed to malathion, corresponding to 1747 with overexpression and 1374 with low expression. In BMMCs exposed to permethrin, 999 genes were found to be overexpressed and 1189 had low expression. Fewer genes with altered expression were observed in PBMCs compared to BMMCs (Figure 1). In cells exposed to malathion, 716 genes were found to be overexpressed and 889 to be underexpressed. BMMCs treated with permethrin showed 748 genes were overexpressed and 823 had low expression. No significant changes in cell viability were detected, all samples showed cell viabilities above 90%.

Two lists of genes were obtained: a list of the total number of genes with changes in gene expression and a list of the 100 genes with the highest and 100 with the lowest expression. Gene ontology analysis was performed on both lists. 

### 2.2. PANTHER GO Analysis with the Complete Lists of Altered Genes 

An analysis with the complete lists of altered genes was performed with PANTHER and included the classifications: “biological process”, “molecular function”, “protein class”, “cell component”, and “signaling pathways”. The over-representation analysis was evaluated by Fisher’s exact test. Supplementary Tables S1–S4 show a summary of the results with statistical significance (*p* < 0.05) according to the over-representation test using the total of the genes. 

**Figure 2:**
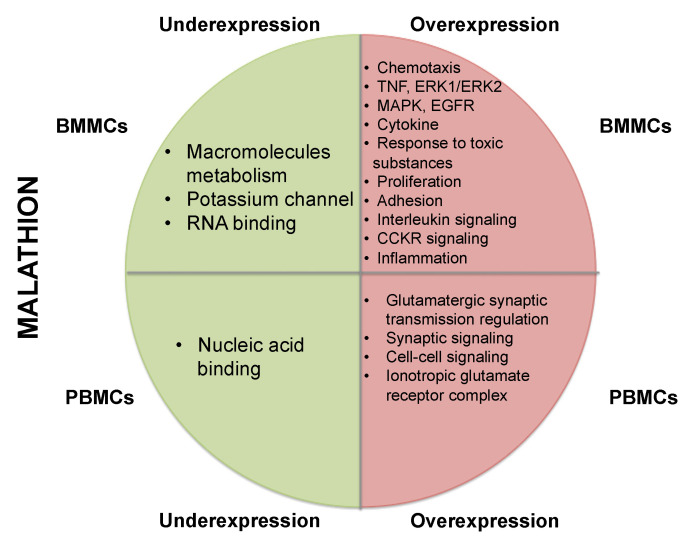
Summary of over-representation test results of genes with altered expression in BMMCs and PBMCs exposed to malathion. Green section shows cellular functions associated with genes with underexpression, and red section with genes with overexpression. The upper part displays results of BMMCs and lower part shows results of PBMCs.

More significant results were obtained in cultures of BMMCs exposed to malathion, mainly when analyzing overexpressed genes. We observed an over-representation of cytokine and chemokine-mediated inflammation function, interleukin signaling, and cholecystokinin receptor (CCKR) signaling (Supplementary Table S1). Additionally, an enrichment of diverse biological processes was observed, including leukocyte chemotaxis, kinase activity regulation, tumor necrosis growth factor cell response (TNF), ERK1/ERK2 cascade positive regulation, MAPK positive regulation, cell communication regulation, epidermal growth factor signaling, cytokine signaling, inflammatory response, and cell adhesion (Supplementary Table S1, Figure 2). In addition, in PBMCs exposed to malathion, we observed that underexpressed genes corresponded mostly to proteins with nucleic acid binding activity. We found that the overexpressed genes participated mainly in biological processes such as glutamatergic synaptic transmission, glutamate receptor signaling, synaptic signaling, and cell–cell signaling (Supplementary Table S2, Figure 2). 

**Figure 3:**
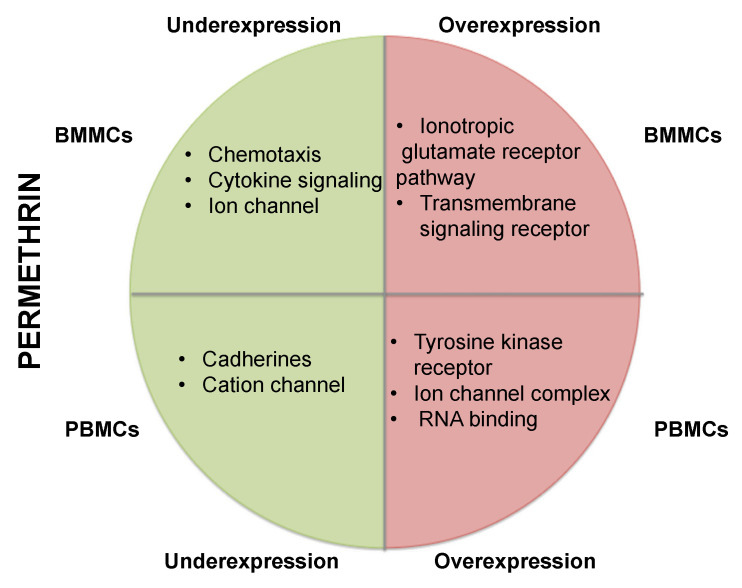
Summary of over-representation test results of genes with altered expression in BMMCs and PBMCs exposed to permethrin. The green section shows cellular functions associated with genes with underexpression and the red section with genes with overexpression. The upper part displays the results of BMMCs and the lower part shows the results of PBMCs.

Analysis of BMMCs exposed to permethrin showed that the underexpressed genes mostly related to cytokines, enzyme binding, and G protein-coupled receptor binding. The most represented biological processes were chemotaxis and cytokine-mediated signaling. In addition, the overexpressed genes corresponded mainly to receptors with transmembrane signaling activity. The most represented pathway was the glutamate ionotropic receptor pathway (Supplementary Table S3, Figure 3). In addition, the analysis of PBMCs exposed to permethrin showed that the underexpressed genes corresponded mainly to cation channels and participated in cadherin signaling, ionotropic glutamate receptor, and Alzheimer–presenilin pathways. Overexpressed genes corresponded mainly to proteins with tyrosine kinase, ion channel, and RNA binding activities (Supplementary Table S4, Figure 3).

**Figure 4:**
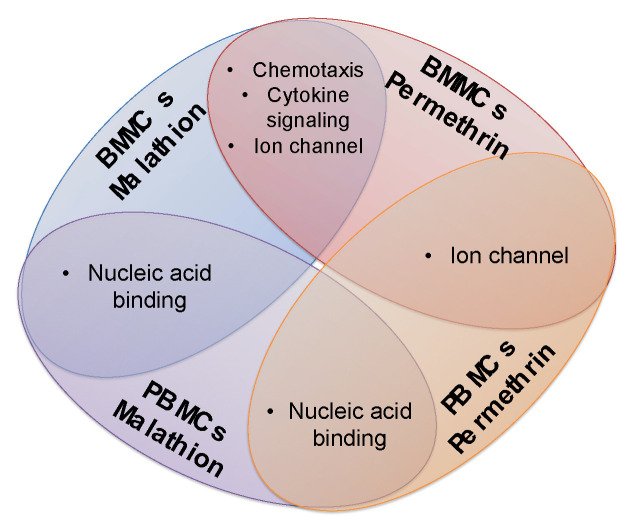
Common cellular functions altered by modification in gene expression in BMMCs and PBMCs exposed to insecticides. Results derived from over-representation test analyzing the total of genes with gene expression changes determined by Panther Pathways program.

Coincidences were observed regarding the most over-represented molecular functions, biological processes, and pathways between BMMCs and PBMCs exposed to the same insecticide. Coincidences between the same type of tissue exposed to a different insecticide were also found (Figure 4). In both types of tissues, malathion deregulated genes that code proteins with nucleic acid binding activity, and permethrin affected the expression of genes that code ion channels. Additionally, we detected that in BMMCs, malathion and permethrin deregulated genes involved in chemotaxis, cytokine signaling, and over-representation of genes coding ion channels were identified. In PBMCs, both insecticides deregulated the expression of genes that code proteins with nucleic acid binding activity (Figure 4). 

### 2.3. PANTHER GO Analysis with the 100 Genes with the Highest Expression and 100 with the Lowest Expression 

**Figure 5:**
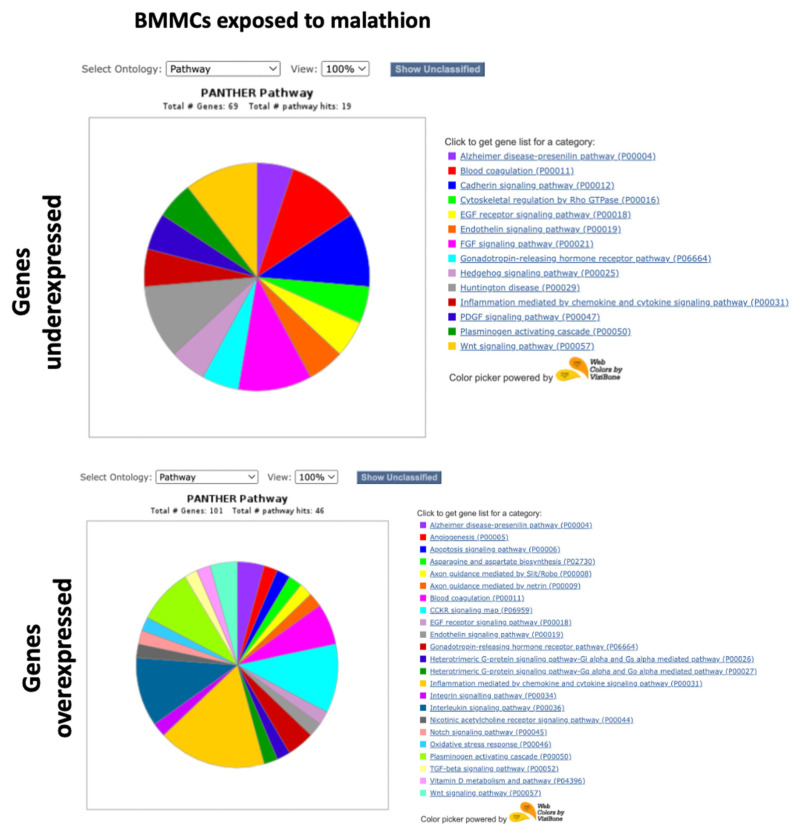
Pathway ontology analysis in BMMCs exposed to malathion.

**Figure 6:**
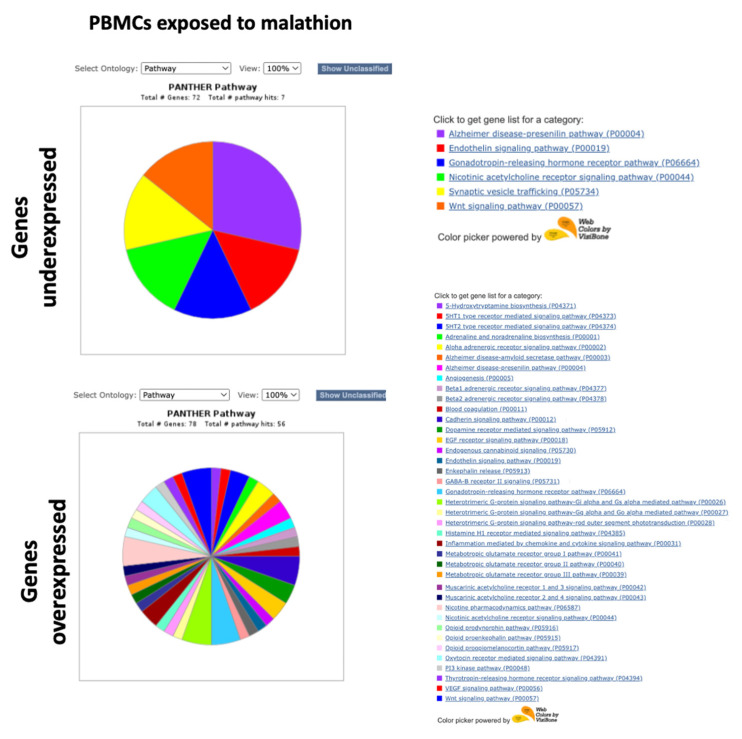
Pathway ontology analysis in PBMCs exposed to malathion.

**Figure 7:**
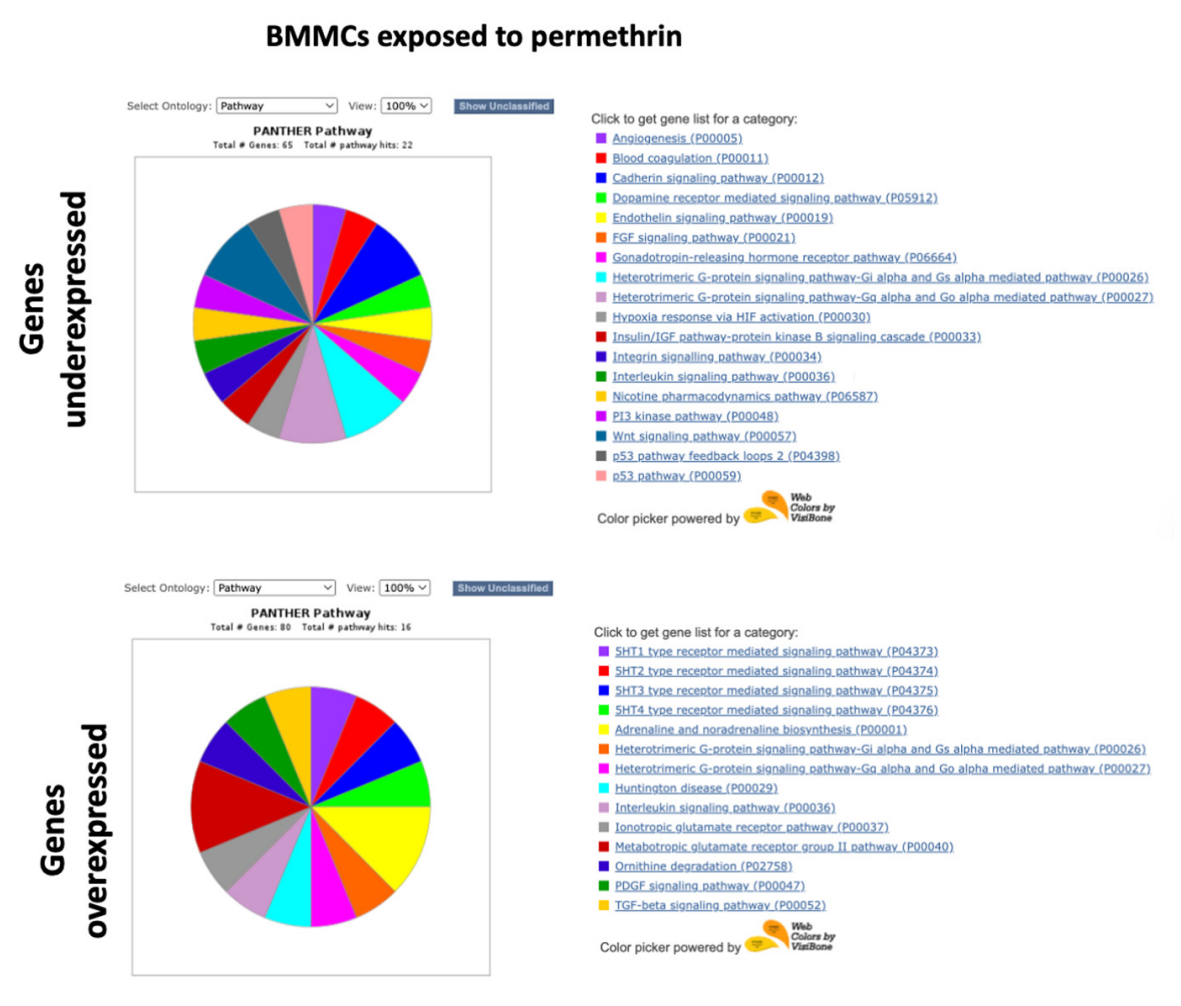
Pathway ontology analysis in BMMCs exposed to permethrin.

**Figure 8:**
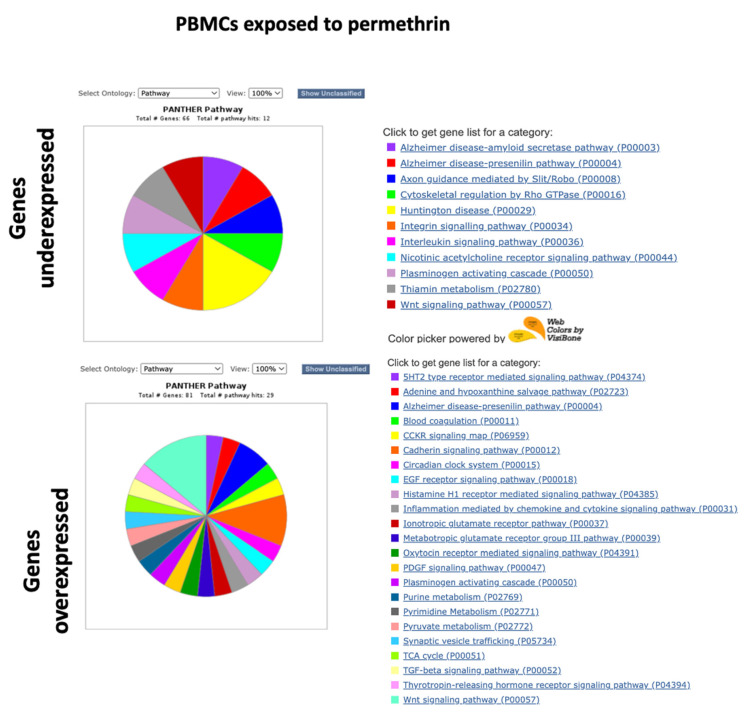
Pathway ontology analyisis in PBMCs exposed to permethrin.

Functional classification was performed with the PANTHER system, using the lists of the 100 genes with the highest expression and the 100 genes with the lowest expression. The “Pathways” ontology was selected and viewed in pie charts. Unclassified genes were filtered. Figure 5, Figure 6, Figure 7 and Figure 8 show the pie charts of the pathways enriched and Supplementary Tables S1–S4 show the lists of genes and their fold changes. Other ontologies analyzed included “Molecular function” and “Biological Process”, Table 1, Table 2, Table 3 and Table 4 show a summary of genes and their functions. 

In the BMMCs exposed to malathion, we found that the genes with the lowest expression coded proteins with DNA binding activity, such as *PROX2* and *NFIB*, and genes that participate in apoptosis, such as *ADAM11* and *ADAM32* (Table 1). Genes underexpressed in BMMCs exposed to malathion participated in pathways such as the endothelin signaling pathway, Wnt signaling pathway, cadherin signaling pathway, inflammation mediated by chemokine and cytokine, and EGF receptor signaling pathway (Figure 5). Among the genes with the highest expression due to exposure to malathion in BMMCs were the cytokines and chemokines *CCL3*, *CCL7*, *CCL19*, *CCL1*, *CCL3L3*, *CXCL8*, *CXCL5*, *CCL1*, *CXCL11*, and *CCL20.* Additionally, genes of the TNF pathway, such as *TNF* and *TNFSF15,* and the transcription factors *HES4*, *NR4A3*, and *NR4A1* were overexpressed in BMMCs exposed to malathion (Table 1). Pathways enriched with genes overexpressed in BMMCs exposed to malathion included inflammation mediated by cytokines and chemokines, the Notch pathway, Wnt pathway, Interleukin signaling pathway, oxidative stress pathway, CCKR signaling map, and TGF-beta signaling pathway (Figure 5). 

Among the genes with the lowest expression detected in the PBMCs exposed to malathion, transcription factors such as *MEIS1*, *ZFP42*, *ARNT2*, and *WBP2NL* were detected, as well as genes associated with cell death, such as *TNFSF15* and *PDIA2,* and genes involved in developmental differentiation, such as *GATA4* and *LEP1* (Table 2). The pathways involved with the genes underexpressed in PBMCs exposed to malathion included the endothelin signaling pathway and Wnt signaling pathway (Figure 6). Among the genes with the highest expression detected in PMBCs exposed to malathion, we found those that code proteins with nucleic acid binding activity, such as *ZFPM2* and *IRX2*; genes that code tyrosine kinase receptors, such as *EPHA10*, *FLT4*, and *ERBB4*, which are involved in the negative regulation of apoptosis; genes associated with epigenetic regulation, such as *FAM172BP* and *MBD2*; and genes of the MAPK pathway, such as *EPHA10*, *FLT4*, *RAPGEF5*, and *ERBB4* (Table 2). A long list of pathways involved with genes overexpressed in PBMCs exposed to malathion were obtained, which included pathways such as cadherin, endothelin, inflammation, VEGF, and Wnt (Figure 6). 

In BMMCs exposed to permethrin, genes with the lowest expression were found to be associated with antioxidant function and stress response, such as *GPX8* and *PROZ*, as well as genes that code nucleic acid binding proteins, such as *TBX15* and *RBMXL2*; genes of the MAPK pathway, such as *FGFR* and *PDGFD*; genes involved in cell death, such as *ADAM30*; genes coding proteins with transcription factor functions, such as *TBX15* and *GATA4*; genes that code sodium channels, such as *SCN9A;* and genes coding proteins with cadherin activity, such as *DCHS2*, *PCDH25*, and *PCDH9* (Table 3). Pathways involved with genes underexpressed in BMMCs exposed to permethrin included the cadherin, FGF, endothelin, interleukin, PI3K kinase, Wnt, and p53 signaling pathways (Figure 7). Genes that code proteins with a nucleic acid function, such as *MBD2*, *FAM172BP*, and *IFIT1*, were found among the genes with the highest expression in BMMCs exposed to permethrin. Additionally, genes associated with apoptosis regulation, such as *BDNF*, genes associated with stress response, such as *FIGF*, genes coding proteins with cytokine activity, such as *IL15R*, and genes of the TGFB pathways, such as *GDF1*, were among the most overexpressed genes in BMMCs exposed to permethrin (Table 3). Pathways enriched in BMMCs exposed to permethrin included the interleukin, PDGF and TGF-beta pathways (Figure 7). 

Among the genes with the lowest expression detected in PBMCs exposed to permethrin were those that code nucleic acid binding proteins, such as *SAMD7*, *LHX3*, *PRX*, and *IFIT1B*; transcription factors, such as *ZFP42* and *WBPNL*; cytokines, such as *A2ML1* and *IL13RA2*; kinases, such as MAPK4, and cell-death-associated genes, such as *LHX3* and *PRUNE2* (Table 4). Pathways enriched included interleukin, nicotinic acetylcholine, and Wnt (Figure 8). Genes with the highest expression detected in PBMCs exposed to permethrin included those that code nucleic acid binding proteins, such as *VGLL3*; cytokines, such as *TNFSF11*; apoptosis-regulation-associated genes, such as *EPHA10*, *EPHA6*, *TNFSF11*, and *PPEF1*; DNA repair genes, such as *HMGA2*; stress response genes, such as *HOXB5*, *FOX11*, *PER3*, *MYT1*, and *KDM5S*; and inflammation-associated genes, such as *PLCH11* (Table 4). Pathways associated with genes overexpressed in PBMCs exposed to permethrin included the CCKR, cadherin, EGF, inflammation, PDGF, ionotropic glutamate receptor, TGF-beta, and Wnt pathways (Figure 8). 

**Figure 9:**
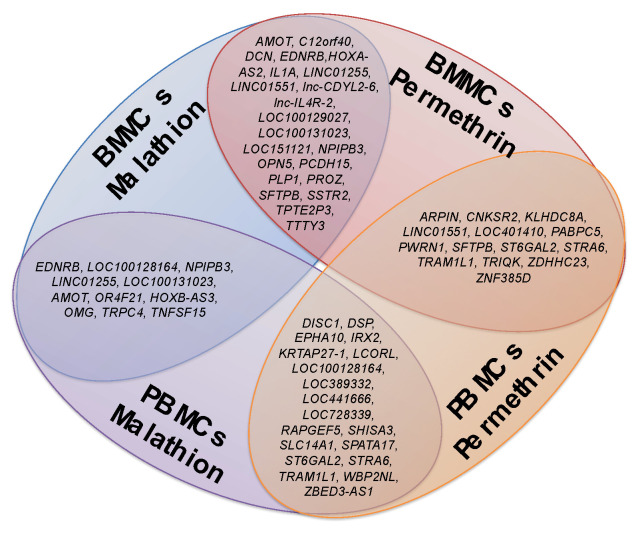
Genes with altered expression in more than one treatment condition and tissue. Results derived from the analysis of the top and bottom 100 genes over or underexpressed in BMMCs and PBMCs, exposed to malathion or permethrin. Figure shows genes that where concordant between two different assays.

Additionally, concordances were found among the altered genes in the different tissues examined, BMMCs and PBMCs, and coincidences among altered genes with the different treatments (malathion and permethrin). Figure 9 shows those genes shared between tissues and treatments.

### 2.4. GeneMANIA Analysis 

**Figure 10:**
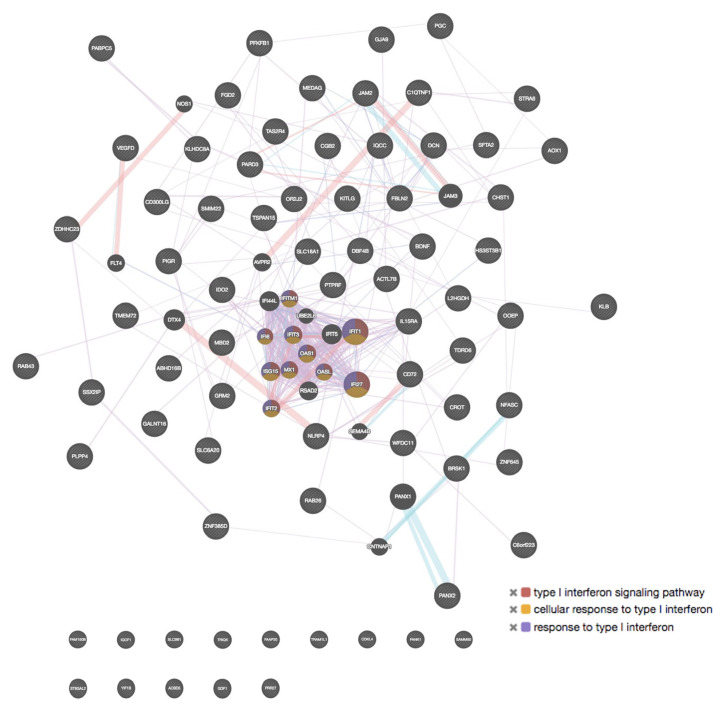
GeneMANIA tool interaction analysis of overexpressed genes in BMMCs exposed to permethrin.

**Figure 11:**
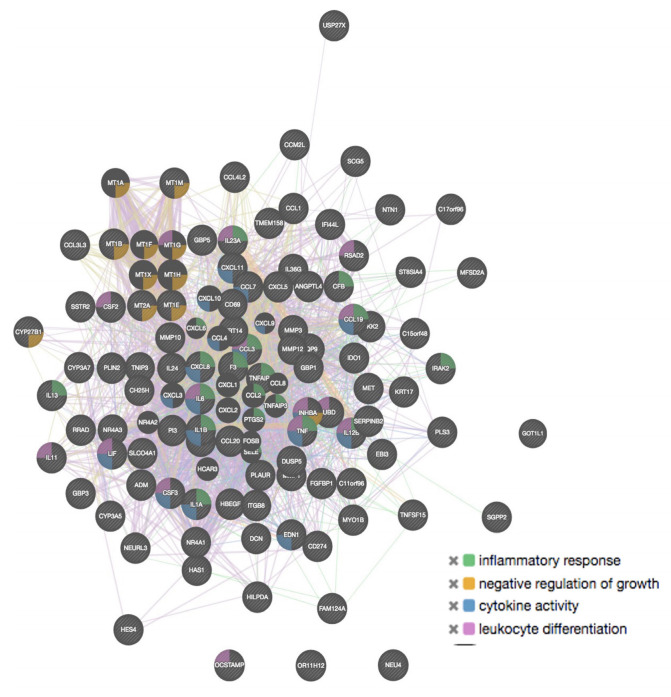
GeneMANIA tool interaction analysis of overexpressed genes in BMMCs exposed to malathion.

To determine the interaction among genes, molecular functions, and common pathways, an additional analysis using the GeneMANIA tool was performed, including the 100 genes with the highest and the 100 with the lowest expression. A summary of the most relevant findings is shown in Table 5 and Figure 10 and Figure 11. Regarding molecular functions, the additional results obtained with GeneMANIA were also detected with the PANTHER tool. In BMMCs exposed to malathion, we found that genes with low expression corresponded to genes associated with ion channel activity that negatively regulate differentiation and cell development. In addition, several high-expression genes are co-expressed and associated with cytokine activity, negative growth regulation, leukocyte differentiation, and inflammatory response; most of these functions were also detected with the PANTHER tool. 

In PBMCs, we found through GeneMANIA analysis that genes with high expression share specific physical interactions and participate in the regulation of synaptic activity. Regarding BMMCs exposed to permethrin, we observed that several genes with elevated expression are co-expressed and participate in type 1 interferon signaling *(IFN1*) (Figure 10). Moreover, in PBMCs, genes with high expression showed genetic interactions associated with functions such as ROS metabolism, ephrin receptors and signaling. 

### 2.5. qPCR Validation

**Figure 12:**
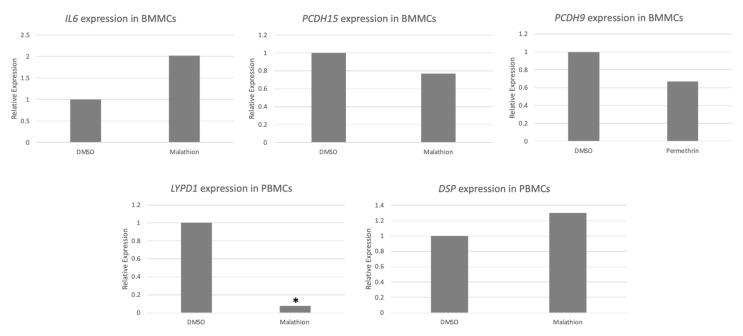
qPCR validation of changes in gene expression in cells exposed to insecticides. Gene expression alterations were assessed on independent PBMCs and BMMCs exposed to malathion and permethrin (0.1 μM, 72 h) * *p* < 0.05, *t* test.

Based on the level of expression and/or relevance in cancer, five genes among the 100 with the highest and 100 with the lowest expression were selected for analysis with qPCR to corroborate microarray expression results. The gene expression of these genes was analyzed on two independent PBMC and BMMC samples from different individuals than those included for the microarrays. The qPCR analysis showed that expression tended to increase or decrease concordantly with the findings of the microarrays. Differences in the degree of increase or decrease were observed between microarray and qPCR analyses, which could be attributed to the differences between both techniques and the use of samples from different individuals. Results were significant for *LYPID,* in which significant underexpression was detected with malathion treatment in PBMCs (Figure 12).

### 2.6. Changes in DNA Methylation of BMMCs Exposed to Insecticides

Considering the results of gene expression obtained with BMMCs, we selected 100 genes for methylation analysis: 25 overexpressed genes and 25 underexpressed that were detected in cells exposed to malathion, and 25 overexpressed and 25 underexpressed that were detected in cells exposed to permethrin. These genes were selected because they were among the 100 with the highest or the 100 with the lowest expression and/or showed relevance for cancer as determined by the ontology analysis. Information was successfully obtained for 78 of the 100 selected genes from the DNA methylation microarray. The complete list of genes is shown in Supplementary Table S9. We analyzed 17 genes with underexpression and 21 with overexpression in BMMCs exposed to malathion. Additionally, we detected 24 genes with underexpression and 16 with overexpression in BMMCs exposed to permethrin. We found that 15.3% (12/78) of genes showed some degree of variation in DNA methylation on the promoter region, which was concordant with gene expression since hypermethylation coincided with gene underexpression and hypomethylation with overexpression. In general, we observed more events of hypomethylation induced by the insecticides. We identified 2/17 (11.7%) genes (*KCNMA1* and *ADAM11*) with hypermethylation in the promoter region in BMMCs exposed to malathion concordant with underexpression results. In addition, we identified 4/21 (19%) genes (*INHBA*, *NR4A3*, *ANGPTL4*, and *ITGB8*) that showed demethylation in the promoter region that matched the overexpression induced by malathion in BMMCS. Regarding permethrin exposure, we found that this insecticide did not induce hypermethylation on the genes analyzed (0/24). On the contrary, demethylation events were frequent, affecting 6/16 (37.5%) of analyzed genes (*ST6GAL2*, *FIGF*, *BDNF*, *PARD3*, *MAPK*, and *TDRD6*), which showed demethylation on the promoter region that was concordant with overexpression in BMMCs exposed to permethrin. Table 6 shows the detected genes in each sample and the corresponding Z values. 

## 3. Discussion

In the present study, we assessed in hematological tissues whether in vitro exposure to permethrin and malathion, two commonly used insecticides, can induce changes in gene expression and DNA methylation, which are events relevant for carcinogenesis [25]. It is important to evaluate whether permethrin and malathion can modify gene expression and if these modifications are due to changes in DNA methylation on promoter regions, since previous studies have demonstrated that environmental agents can alter DNA methylation, which can modify gene expression [13,14,26,27,28]. Moreover, in populations exposed to certain pollutants, such as ultrafine carbon particles and endocrine disruptors, gene expression and methylation have been assessed in PBMCs and characteristic expression profiles have been detected, which are considered to be footprints evidencing the exposure to these specific chemicals [29,30]. 

Different classes of insecticides can modify DNA methylation and expression of genes coding cytokines, chemokines, transcription factors, and signal transductors [26,27,31,32]. Although studies that have evaluated the effect of pyrethroids are very limited, it has been shown that these insecticides can alter the expression of genes such as *WNT10B*, *THBS1*, *SPP1*, *FEZ1*, and *GPNM*. Given the very scarce information regarding the effect of permethrin and malathion on gene expression, we aimed to evaluate in vitro whether these insecticides alter gene expression and methylation in PBMCs and BMMCs, which are hematopoietic tissues that can adequately reflect the scenario where hematological malignancies occur. 

Our results showed that both insecticides modify global gene expression in PBMCs and BMMCS exposed in vitro; different results were obtained between these tissues. In general, more alterations in gene expression were observed in BMMCs, which suggests that more undifferentiated and immature cells show more susceptibility to the insecticides. In populations exposed to pesticides, it is more common to study PBMCs because of the feasibility of obtaining the sample and because it is not as invasive as other tissues [26]. However, in this study, we observed that tissues other than PBMCs could show higher susceptibility, such as BMMCs. Thus, studies may underestimate the effects of pesticides at the transcriptional level if only PBMCs are studied. On the other hand, it is important to consider that one limitation of this study is that we included PBMCs and BMMCs from different donors, and differences seen among these tissues could be related to individual biological variability. Unfortunately, in the present study we were not able to include PMBCs and BMMCs from the same donor. 

The concentrations of permethrin and malathion used in this study (0.1 µM) are concentrations with biological relevance. We considered that it was of importance to include concentrations reflecting scenarios of human exposure [20]. In addition, the time of exposure of 72 h was selected as it was considered to be an optimal time for the cell culture of PMBCs and BMMCs without having cell viability decrease. We decided to apply the insecticides every 24 h to maintain the 0.1 μM concentrations and to simulate a subchronic exposure. 

### 3.1. Underexpression of Genes in BMMCs Exposed to Malathion

Among the genes found to be repressed with malathion exposure in BMMCs, *KCNMA1*, which is associated with hypokalemia and paralysis, was detected [33,34]. We observed an over-representation of pathways involved in neuronal function, which was expected because this is a common effect of organophosphate pesticide. Several potassium channels were repressed. Besides the effect of malathion on genes associated with neurotoxicity, we detected the repression of genes relevant to cancer onset and development (Supplementary Table S11). We found low expression of the disintegrin and metalloproteinase families of the genes *ADAM11* and *ADAM32*, which have relevance in cancer [35]. An additional family of genes repressed in BMMCs exposed to malathion is the protocadherin family. Hypermethylation of different members of the protocadherin family has been reported in cancer [36,37]. In addition, we detected repression of the *EDNRB* gene in BMMCs exposed to malathion, which suffers hypermethylation in different types of leukemia [38].

### 3.2. Overexpression of Genes in BMMCs Exposed to Malathion

Gene ontology analysis revealed that among genes with high expression in BMMCs exposed to malathion were genes coding cytokines and chemokines, proteins associated with processes such as inflammation, oxidative stress response, and response toxic substances, as well as pathways such as *MAPK*, Wnt, *TNF*, and Notch were over-represented. Additionally, the Notch and Wnt pathways were enriched when analyzing genes with the highest expression. One important finding was the induction of expression of genes involved in the inflammatory response, such as *IL6*, *IL1B*, *CXCL8*, *CCL3*, *CCL7*, *CCL3L3*, and *CCL20*. This result was exclusively seen in BMMCs treated with malathion, given that in PBMCs, a lower number of genes associated with inflammation was observed. Previous studies have reported that organophosphate exposure modifies chemokines and cytokines, concordant with our findings [39]. This study adds to earlier reports that show that exposure to low levels of organophosphates induces an inflammatory response [40]. Although the mechanism by which organophosphates trigger the inflammatory response has not been elucidated, it is suggested that these insecticides interact directly with inflammatory cells and trigger the release of inflammation mediators. It has been reported that exposure to malathion stimulates macrophages for generating ROS and cathepsin D, can potentiate phagocytosis and antigen presentation, and induces the release of histamine by mast cells and basophils. Among the overexpressed genes that encode pro-inflammatory cytokines, *IL6* was detected, which is a relevant finding because it has a role in leukemogenesis (Supplementary Table S11) [41]. Together with the increase in this cytokine, we found an increase in *TNFα* and *IL1A* expression, which are signals that promote the production of *IL6* [41,42]. This suggests that in BMMCs, the effect of malathion induces a pro-inflammatory environment by increasing the levels of TNF and IL1A, which lead to an increase in *IL-6*. The increase in *TNF*-α expression as an effect of malathion is a relevant finding because this cytokine participates in all the steps of leukemogenesis and is overexpressed in pre-B ALL [43]. It is thus relevant to study the role of malathion exposure on TNF deregulation to a greater extent in other models (Supplementary Table S11). 

Additionally, among the pathways with more over-representation, we detected MAPK, ERK1/ERK2, NFkB, Notch, Wnt, and JNK, which are involved in different cell functions such as proliferation, survival, differentiation, maturation, angiogenesis, inflammatory response, and migration, and have a very important role in hematopoiesis; their participation in leukemia and other types of cancer has been reported [44,45,46,47,48]. 

### 3.3. Gene Expression Deregulation in PBMCs Exposed to Malathion

We detected that genes with underexpression corresponded to nucleic acid binding proteins, such as the transcription factor ZFP42. *ZFP42* (*REX1*) regulates the proliferation of human mesenchymal cells through MAPK signaling [49]. In PBMCS, the expression of the *MAP4K3* gene was increased; however, it remains to be investigated if the MAPK pathway deregulation occurs through the inhibition of *REX1* [43], which seems to be repressed by malathion (Supplementary Table S11). Additionally, we observed underexpression of the *MEIS1* gene. The repression of this gene is associated with higher levels of ROS. Malathion and other organophosphates induce ROS, and this phenomenon could be potentiated through the inhibition of *MEIS1* expression [50,51]. As in BMMCs, the expression of the *EDNRB* gene was observed to be lowered in PBMCs exposed to malathion, which shows that in both tissues, this gene is repressed by the effect of this insecticide. This gene has shown relevance in cancer (Supplementary Table S11) [38]. 

In PBMCs exposed to malathion, the overexpressed genes were involved in glutamate receptor signaling, synaptic signaling, and cell–cell signaling; the pathway with the highest over-representation was the cadherin pathway. We detected overexpression of the *GRIK2* gene, which is considered a tumor suppressor gene [52,53] (Supplementary Table S11). Like BMMCs, in PBMCs exposed to malathion, we identified overexpression of the Wnt pathway, which is vital in leukemia and lymphoma [54,55]. In addition, we observed the deregulation of genes involved in the cadherin pathway, apoptosis regulation, epigenetic deregulation, and inflammation; these processes have a crucial role in carcinogenesis (Supplementary Table S11) [43]. 

### 3.4. Gene Repression in BMMCs Exposed to Permethrin

With this tissue and agent, we identified a significant over-representation of repressed genes coding proteins with cytokine and chemokine function. It has been previously suggested that insecticide exposure can have an immunotoxic effect, modifying the activity of specific cell types and altering cytokine secretion [31]. This study agrees with previous reports because we detected expression modification in several genes associated with cytokine and chemokine functions. Moreover, these molecules show repressed expression in certain types of cancer. We found repression of the *CXCL8* chemokine with permethrin exposure; this is an important finding because this gene can have an antitumor role [56]. However, this contrasts with the low expression of other cytokines and chemokines, such as *CXCL12*, that have a pro-tumoral effect. Among cytokines with a low expression, we found the *OSM* gene, which has the potential to inhibit myeloid leukemic cell proliferation [57]. In addition, we observed that permethrin represses the expression of the *TGFB2* gene in BMMCs, which has a tumor suppressor function (Supplementary Table S11) [58,59]. 

Similarly, we detected low expression of the *GPX8* gene in BMMCs exposed to permethrin. GPx proteins regulate oxidative stress. The low expression of *GPX8* may enable permethrin toxicity through the accumulation of ROS and lipid peroxidation [60]. Another gene that showed repression was *GATA4*, which has been recognized as a tumor suppressor gene in different types of cancer [61]. In addition, we detected a high representation of genes repressed encoding ion channels such as *SCN2A*, *SCNN1*, *SCN9A*, and *SCN3B*, which encode voltage-dependent sodium channels. Our study shows that pyrethroids can alter ion channels at the gene expression level [62]. Additionally, we found the repression of genes coding protocadherins, such as *PCDH15* and *PCDH9*; the deregulation of these genes is a characteristic of leukemic cells (Supplementary Table S11) [43]. 

### 3.5. Gene Overexpression in BMMCs Exposed to Permethrin

Among the genes with overexpression, we detected mainly genes of the ionotropic glutamate receptor pathway. This is concordant with previous reports and confirms that certain neuroactive insecticides, such as pyrethroids, act over ionic channels, including the ionotropic glutamate receptor [63]. Overexpression was also detected for the *MBD2* nucleic acid binding gene, which can promote DNA demethylation [64]. An important finding was the overexpression of the *IFIT1* gene in BMMCs exposed to permethrin; overexpression of this gene has been detected in myelodysplastic syndromes before the development of leukemia [65], and is relevant for pre-B ALL (Supplementary Table S11) [66]. IFIT1 is modulated by the JAK/STAT pathway and is an inflammation related protein that can be aberrantly expressed in cancer. The overexpression of *IFIT1* was also highlighted with the Genemania analyis, in which response to IFN1 pathway was observed. IFITs are quickly induced by multiple stimuli, such as IFN dependent or IFN independent pathways and have been linked to cancer [67]. Our data agrees with previous reports that showed that the pyrethroid ivermectin induces expression of *IFIT1*, *IFIT2*, *IF144*, *ISG20*, *OASL* and *IRF9* [68].

### 3.6. Gene Repression in PBMCs Exposed to Permethrin

We detected low expression of genes coding cadherins, sodium channels, and genes associated with Alzheimer–presenilin signaling (Supplementary Table S11). Some genes coding cadherins participate in the Wnt pathway, which is relevant in hematopoiesis and leukemogenesis [47]. Previous studies suggest that pyrethroids can alter Wnt pathway genes (*WNT10*) [69]. In this study, we found deregulation of members of the Wnt pathway family (*WNT2*, *WNT4*, *WNT7A*). Among the repressed genes, we found a high number of genes involved in cadherin signaling, such as *CADH19*, *TCF7L2*, and protocadherins *PCDH9*, *PCDH15*, *PCDH12*, *PCDH114*, and *PCDHGA5*. Protocadherins are cell–cell adhesion proteins that participate in aging and cancer. [70]. Additionally, repression of *PRUNE2*, which participates in cancer, was detected in PMBCs exposed to permethrin [71]. 

In PBMCs exposed to permethrin, we also detected repression of genes that participate in Alzheimer’s development, such as the metalloproteins *MMP12*, *MMP2*, and *MMP13*, among others [72]. Additionally, we found deregulated genes coding cationic channels, which have also been associated with the neurotoxicity of permethrin. This study provides relevant evidence showing that permethrin and malathion have a strong effect on genes involved in the central nervous system, which was evident even in cells of hematopoietic origin. It is possible that the topology of the regions in which the affected genes are located makes them particularly sensitive to the effect of insecticides. It is also possible that genes coding transcription factors associated with neurological processes were deregulated. In fact, with both insecticides, we found an over-representation of genes coding proteins with nucleic acid binding activity, many of which were transcription factors. We also found correlations among genes deregulated by malathion and permethrin, suggesting that although having different chemical structures, these chemicals can share mechanisms of action, which has already been reported [73]. Although metalloproteins are mainly involved in the central nervous system, the results obtained in this study and those described in the literature suggest that they can also participate in other processes, including cancer [74]. 

### 3.7. Overexpression of Genes in PMBCs Exposed to Permethrin 

The over-representation analysis showed that the genes with increased expression were significantly associated with the vasopressin synthesis pathway, transmembrane receptors functions, ion channels, and nuclear processes. Regarding vasopressin synthesis, overexpression of the *NEU2* gene was evidenced. It is reported that overexpression of *NEU2* impedes cell growth, blocking different cell cycle checkpoints and affecting all cell cycle phases [75] (Supplementary Table S11). We also detected a significant over-representation of genes coding proteins with transmembrane receptor function, including *ROS1*, *KIT*, and *ALK*, which showed overexpression in PBMCs exposed to permethrin. These genes belong to the tyrosine kinase receptor family and have been identified as oncogenes in diverse types of cancer [76,77,78]. 

### 3.8. Methylation Alteration in BMMCs Exposed to Insecticides

We observed that both malathion and permethrin induce changes in gene expression. Organophosphates are reported to cause changes in DNA methylation [13]. Concordant with previous studies, we found that malathion can alter DNA methylation, and those changes matched changes in gene expression. These relevant findings support studies on exposed farmers showing changes in DNA methylation due to exposure to organophosphate mixtures [79]. We found that permethrin does not induce DNA hypermethylation; however, demethylation events were very frequent, matching the overexpression of the affected genes. However, we also found repressed genes, in which no hypermethylation on promoter region was detected, suggesting that permethrin can lead to gene silencing through alternative mechanisms different from DNA methylation. Although the effect of pyrethroids on DNA methylation has not been widely studied in human beings, murine models have reported that permethrin can induce a decrease in DNA methylation, which is concordant with our findings [28,80]. It is proposed that permethrin could induce DNA demethylation through mechanisms dependent on ROS. Previous studies have not shown that changes in DNA methylation induced by permethrin match with changes in the expression of DNMTs (DNA methyltransferases). 

The present study found higher expression on DNMTs, and thus the mechanism involved remains to be elucidated. An alternative to partially explain demethylation induced by permethrin is through the *MBD2* gene. We detected that permethrin could induce *MBD2* gene expression, which is associated with active DNA demethylation. MBD2 can directly remove the methyl group through hydrolysis [81]. Additionally, in PBMCs exposed to permethrin, we found overexpression of the *ROS1* gene, which encodes a DNA glycosylase that participates in DNA demethylation through the base excision DNA repair pathway [81]. The induction of *ROS1* overexpression represents a possible pathway by which permethrin increases DNA demethylation and another possible mechanism is the activation of *RELN*, whose activation has been associated with demethylation in rat neurons [81]. In our study, we detected overexpression of the *RELN* gene in PBMCs exposed to permethrin that could contribute to DNA demethylation events.

## 4. Materials and Methods

### 4.1. Cell Culture and Treatment

Informed consent was obtained according to the recommendations of the Helsinki Declaration for all samples. The project (INP 001/2013) was approved and followed the guidelines of Research and Institutional Ethics. A 30 mL peripheral blood sample was acquired from two young, healthy, non-smoker male donors. Two 10 mL bone marrow samples, left over from the samples used for transplants, were obtained. Mononuclear cells were isolated from peripheral blood or bone marrow using a density gradient medium (Lymphoprep, Serumwerk, Bernburg, Germany) and cultured at a final density of 5–7 × 10^5^ cells/mL in triplicate, in RPMI1640 medium (Thermo Fisher Scientific, Waltham, MA, USA) supplemented with 10% fetal bovine serum (Thermo Fisher Scientific, Waltham, MA, USA), 1% non-essential amino acids (Thermo Fisher Scientific, Waltham, MA, USA), 1% sodium pyruvate (Thermo Fisher Scientific, Waltham, MA, USA), and 1% L-glutamine (Thermo Fisher Scientific, Waltham, MA, USA). Cells were cultured for 72 h at 37 °C and 5% CO_2_ and were treated at 0, 24, and 48 h after culture initiation with permethrin and malathion at a final concentration of 0.1 μM. Treatment with 0.2% DMSO (Merck, Darmstadt, Germany) was a negative control because it dissolved the insecticides. Cell viability was assessed with Trypan blue (Merck, Darmstadt, Germany) exclusion assay at the beginning and the end of cell culture (0 and 72 h). 

### 4.2. Nucleic acid Extraction and Microarrays

After 72 h of culture, cells were washed with PBS 1× (Thermo Fisher Scientific, Waltham, MA, USA) and cell viability was determined by trypan blue exclusion assay. At least 1 × 10^6^ cells were separated in 300 μL of TRIzol (Thermo Fisher Scientific, Waltham, MA, USA) for RNA extraction according to the manufacturer’s guidelines. The remaining cells were used for DNA extraction using Qiamp DNA midi Kit (Qiagen, Hilden, Germany). Pools of equal parts of cells in TRIzol to obtain RNA or equivalent amounts of DNA from each donor were prepared for the microarrays. A microarray containing pools of RNA (gene expression microarray) or pools of DNA (DNA methylation microarray) was performed for each treatment condition and tissue. Agilent “Human GE 4×44K v2 Microarray” (G4845A) was used for gene expression analysis (Agilent Technologies, Santa Clara, CA, USA). In each PBMCs or BMMCs gene expression microarray, cRNA from cells exposed to insecticide (permethrin or malathion) were hybridized together with cRNA from cells exposed to solvent (DMSO), according to Agilent USA protocol. Briefly, after RNA extraction, amplification and labeling with fluorochrome were performed to generate cRNA Cy5 or Cy3 using a Low Input Quick Amp Labeling two-color kit (Agilent Technologies, Santa Clara, CA, USA) in the presence of a two-color spike-in control. After purification, labeled cRNA was hybridized onto “Human GE 4×44K v2 Microarray, “containing RNA sequences of 27,958 genes from the Entrez Gene database. Scanning was performed with an Agilent scanner G2505, and data extraction was performed with Feature Extraction Software Scan Control 8.3 [82]. Data were analyzed and processed with the Gene Spring GX 14.9 program (Agilent Technologies, Santa Clara, CA, USA).

DNA methylation analysis was carried out with 6–12 μg of DNA of each BMMCs sample exposed to insecticides or solvent. Then, sonication and immunoprecipitation of methylated DNA were performed. DNA was washed and purified with a mixture of phenol:chloroform:isoamyl alcohol and was labeled with Cy5. Control genomic DNA was labeled with Cy3. Both immunoprecipitated and control genomic DNA were hybridized on the “Human DNA Methylation Microarray” (G4495A-023795) 1×244K (Agilent Technologies, Santa Clara, CA, USA), which covers 27,672 CpG islands of promoter and non-promoter regions. Finally, washing, scanning, and data extraction were performed according to the manufacturer’s recommendations [83]. Data analysis was performed with the Agilent Workbench 7.0 program.

### 4.3. Data Analysis 

Gene expression was analyzed with Gene Spring GX 14.9 Agilent Software (Agilent Technologies, Santa Clara, CA, USA), which was used to obtain lists of genes that had a >2.0-fold change in gene expression compared to the control. One hundred genes with the highest and one hundred with the lowest expression were selected. Using the total number of genes with >2.0 fold change and with the top and bottom 100 genes, molecular function, biological processes, cellular components, protein classes, and pathways were analyzed with PANTHER PATHWAYS 14.1 (Protein Analysis Through Evolutionary Relationships), which belongs to the Gene Ontology Reference Genome Project (GO) [84]. The PANTHER tool was used to analyze the distribution of each category of ontology. The over-representation test tool was used for statistical analysis, comparing the list of genes of interest with the reference list using the Fisher test and determining if a particular class of genes was over-represented. A *p* < 0.05 was considered significant [85]. Additionally, interaction among the 100 genes with higher expression and the 100 with the lowest gene expression was achieved with the GeneMANIA web tool [86]. 

Considering the expression data obtained, 78 genes were analyzed for changes in DNA methylation with Workbench 7.0 Agilent software, comparing samples exposed to insecticides with samples exposed to DMSO (Supplementary Table S5). Z factors of each gene were compared, considering a high or positive Z factor to be methylated DNA and a low or negative Z factor to be non-methylated DNA. The analysis focused on methylation changes in promoter regions, considering DNA methylation status and gene expression concordance. The sum of the Z values of all the probes in the promoter region of the gene of interest exposed to insecticides was compared to the sum of the Z values of all the probes in the promoter region of the gene of interest exposed to DMSO. Differences between the sum of Z values were considered when higher than 20. 

### 4.4. qPCR Validation 

From the list of genes with significant fold changes in gene expression, five were selected for qPCR assay in additional independent PBMC and BMMC samples from different volunteers than those included in the microarray. Relative quantification of the gene expression levels of the transcripts of *PCDH15*, *PCDH9*, *IL6*, *DSP*, and *LYPD1* genes was acquired on a LightCycler 2.0 (Roche Applied Science, Penzberg, Germany) (Supplementary Table S10). Cell cultures were conducted as described before. At 72 h after cell culture, the viability with trypan blue exclusion was analyzed, and RNA extraction was carried out with the Trizol (Thermo Fisher Scientific, Waltham, MA, USA) method. RNA integrity was verified on an agarose gel, and RNA quantification was assessed on an Epoch (BioTek, Winooski, VT, USA). cDNA was obtained with the Transcriptor First Strand cDNA kit (Roche, Penzberg, Germany). LightCycler probes from Universal Probe Library System (Roche, Penzberg, Germany) were used for qPCR amplification. Primers and probes used for each gene are described in Supplementary Table S10. Transcript quantification was calculated in duplicates with the ΔΔCT method, using the *GUSβ* gene as endogen control for data normalization. Statistical analysis was performed with a T-test, with a *p* < 0.05 considered significant.

## 5. Conclusions

We observed that malathion and permethrin induce several gene expression modifications in both tissues. Notably, many genes showed changes in expression in BMMCs exposed to malathion. Through gene ontology analysis, we found that permethrin deregulates ion channels in PBMCs and BMMCs, and malathion alters genes coding proteins with nucleic acid binding capacity, which was also observed in PBMCs exposed to permethrin. Additionally, we found that both insecticides deregulated genes with chemotaxis functions, ion channels, and cytokines. Several genes deregulated in this study are potentially associated with cancer onset and development, and some of them have been reported to be deregulated in hematological cancer. It is important to take into consideration that the cellular model used here included cells without malignant transformation and our model was limited to study changes in gene expression and methylation; further studies confirming if these events could drive leukemogenesis are needed. In addition, we detected that both pesticides could induce DNA methylation changes that matched changes in gene expression. We found that permethrin does not induce hypermethylation but can induce hypomethylation, and malathion generated both phenomena. These results suggest that both pesticides have the potential to modify gene expression through changes in promoter DNA methylation and probably through other mechanisms that should be investigated. Our study shows that permethrin and malathion induce biological changes that could be associated with diseases such as cancer. Further studies, including those using animal models, are needed to assess whether these results are also observable in vivo. 

## Figures and Tables

**Table 1:** Highlights of gene ontology analysis of the top and bottom 100 genes with over and underexpression in BMMCs exposed to malathion.

BMMCs Exposed to Malathion		
	Underexpression	Overexpression
Molecular Function	Structural, cytoskeleton: *TUBB8*	Cytokines: *CCL3*, *CCL7*, *CCL19*, *CCL1*, *CCL3L3*, *CCL20*
		Cytokine receptor binding: *IL23A*
		Hormonal receptor binding: *ADM*, *SCG5*, *EDN1*
Biological process	Cell growth: *LINGO1*, *LRIG3*	Negative regulation of apoptosis: *MET*
	Cell differentiation: *PLP1*	
	Cell death: *ADAM11*, *ADAM32*	
	Mesoderm development: *PITX1*, *FGF22*	
	Stimulus response: *OPN5*, *LINGO1*, *LRIG3*, *DDR1*, *OR4D1*, *CEACAM7*, *OR2AT4*, *OR4F29*, *PROZ*, *OR4F21*, *AMOT*, *OR6C2*, *OR9G4*	
Protein class	Defense/Immunity: *CRISP3*	Adhesion: *ITGB8*, *ANGPTL4*
		Growth factor: *INHBA*, *HBEGF*
	Signaling: *PF4V1*, *FGG*	Chemokines: *CXCL8*, *CCL19*, *CXCL5*, *CXCL11*
	Transcription factor: *PROX2*, *NFIB*	Tumoral necrosis factor family: *TNF*, *TNFSF15*
		Transcription factor: *HES4*, *NR4A3*, *NR4A1*

**Table 2:** Highlights of gene ontology analysis of the top and bottom 100 genes with over and underexpression in PBMCs exposed to malathion.

PBMCs Exposed to Malathion		
	Underexpression	Overexpression
Molecular Function	Methyltransferase: *HNMT*	Protein kinase: *CDK15*, *GRK1*
Biological Process	Cell cycle: *S100A1*, *CROCCP3*	Negative regulation of apoptosis: *EPHA10*, *FLT4*, *ERBB4*
	Development differentiation: *GATA4*, *LEP1*	Epigenetic regulation: *FAM172BP*, *MBD2*
	Immune response: *CMA1*	Signals transduction: *RAPGEF5*
	Stress response: *APLF*, *PDIA2*	Differentiation during development: *GP5*
		Embryonic development: *DLG2*
		DNA replication: *GMNC*
Cell component	Intracellular junctions: *AMOT*	Nucleus: *TCF7L1*
	Microtubules: *KIF6*	
Protein class	Cytokines: *TNFSF15*, *SST*	Adhesion: *RS1*
		Nuclease: *TDRD6*, *TDRD1*
		Transcription factor: *IRX2*, *ZFPM2*
	Transcription Factor: *MEIS1*, *ARNT2*, *ZFP42*, *WBP2NL*, *EN1*	
Pathway	Endothelin: *EDNRB*	Inflammation: *PLCB4*, *GNAI1*
		Wnt: *PCDHGB1*

**Table 3:** Highlights of gene ontology analysis of the top and bottom 100 genes with over and underexpression in BMMCs exposed to permethrin.

BMMCs Exposed to Permethrin		
	Underexpression	Overexpression
Molecular Function	Antioxidant: *GPX8*	Nucleic acid binding: *IFIT1*
		Hormonal activity: *CGB2*
Biological process	Negative regulation of apoptosis: *FGFR2*	Cell adhesion: *ZNF645*, *PARD3*, *SSX21P*
		Negative regulation of apoptosis: *BDNF*
		Epigenetic regulation: *FAM172BP*, *MBD2*
		Chromatin organization: *MBD2*
	Cell cycle: *MAPRE3*, *TPTE2*	Signal transduction: MAPK: *GDF1*
		Cell cycle: *CDKL4*, *ACTL7B*
	Differentiation during development: *FGFR2*, *HMCN2*, *BSPH1*, *PLP1*, *GATA4*, *LMOD1*	Differentiation during development: *DCN*, *GDF1*, *BDNF*, *BRSK1*
	Cell death: *ADAM30*	Cell Death: *IFI27*
	Stress response: PROZ	Stress response: *FIGF*
Cell component	Nucleus: *CSN3*, *RBMXL2*	Nucleus: *CDKL4*
Protein class	Adhesion: *ITGB6*	Adhesion: *FBLN2*
		Nuclease: *TDRD6*
	Transcription factor: *TBX15*	Growth factors: *BDNF*
	Voltage dependent sodium channel: *SCN9A*	
Pathways	Angiogenesis: *PDGFD*	Interleukins: *IL15RA*
	Cadherins: *DCHS2*, *PCDH15*, *PCDH9*	
	Endothelin: *EDNRB*	
	PI3K pathway: *TPTE2*	
	P53: *TPTE2*	

**Table 4:** Highlights of gene ontology analysis of the top and bottom 100 genes with over and underexpression in PBMCs exposed to permethrin.

PBMCs Exposed to Permethrin		
	Underexpression	Overexpression
Molecular Function	Nucleic acid binding: *PRX*, *IFIT1B*	
	Transcription factor: *ZFP42*, *WBP2NL*	
	Cytokines: *A2ML1*, *IL13RA2*	
Biological Process	Negative regulation of apoptosis: *LHX3*	Chromatin ensemble: *HIST2H3BF*
	Cytoskeleton organization: *TUBB8*	MAPK cascade: *EPHA10*, *RAPGEF5*, *EPHA6*, *GAB1*
	Cell cycle: *SAMD7*	Differentiation during development: *SPRR1A*
	Cell Growth: *MXRA5*, *SEMA5B*	
	Differentiation during development: *PSAPL1*	Cell death: *TNFSF11*, *PPEF1*
		DNA repair: *HMGA2*
	Cell death: *PRUNE2*	Splicing: *RBMX2*
	Mesoderm development: *COL8A1*	Cytokine production: *BTNL3*
		Stress response: *ATG10*
Cell component		Nucleus: *PUS7L*, *VGLL3*
Protein Class	Kinases: *MAPK4*	Transcription factor: *HOXB5*, *FOXI1*, *PER3*, *MYT1*, *KDM5A*
Pathways	Wnt: *ANKYRIN*	Inflammation: *PLCH11*
		Wnt: *PCDHA8*, *PCDHB18P*, *MMTV*, *WNT11*, *MMP7*

**Table 5:** GeneMANIA analyses of the 100 genes with the highest overexpression and the 100 with the lowest under expression. (-) No results were obtained.

		Underexpression	Overexpression
Malathion	BMMCs	Channel activity	Inflammatory response
		Negative regulation of cell development	Growth negative regulation
		Ion channel activity	Cytokine activity
		Cell differentiation negative regulation	Leukocyte differentiation
	PBMCs	-	Synaptic regulation activity
Permethrin	BMMCs	-	Type 1 Interferon signaling pathway
			Type 1 Interferon cellular response
	PBMCs	Wide pore cannel activity	Reactive oxygen species regulation
			Ephrins signaling
			Protein-protein interaction, bridging

**Table 6:** Methylation analysis of selected genes.

**Repressed Genes in BMMCs Exposed to Malathion**					
**Gene**	**Locus**	**ΣZ DMSO**	**ΣZ Malathion**	**Methylation Status**	**ΣZ Malathion**- **ΣZ DMSO**
*KCNMA1*	10q22.3	39.65	62.97	Hypermethylated	23.32
*ADAM11*	17q21.31	−44.58	55.14	Hypermethylated	99.72
**Overexpressed genes in BMMCs exposed to malathion**					
**Gene**	**Locus**	**ΣZ DMSO**	**ΣZ Malathion**	**Methylation status**	**ΣZ DMSO-** **ΣZ Malathion**
*INHBA*	7p14.1	9.74	−11.36	Hypomethylated	21.10
*NR4A3*	9q22	167.30	65.15	Hypomethylated	102.15
*ANGPTL4*	19p13.2	31.23	−62.18	Hypomethylated	93.41
*ITGB8*	7p21.1	66.01	0.25	Hypomethylated	65.77
**Overexpressed genes in BMMCs exposed to permethrin**					
**Gene**	**Locus**	**ΣZ DMSO**	**ΣZ Permethrin**	**Methylation status**	**ΣZ DMSO-** **ΣZ Permethrin**
*ST6GAL2*	2q12.3	86.51	−3.67	Hypomethylated	90.17
*FIGF*	Xp22.2	28.40	0.92	Hypomethylated	27.48
*BDNF*	11p14.1	128.06	−1.27	Hypomethylated	129.32
*PARD3*	10p11.22-p11.21	27.95	−5.93	Hypomethylated	33.88
*MAPK*	22q11.22	18.07	−13.69	Hypomethylated	31.76
*TDRD6*	6p12.3	62.78	0.03	Hypomethylated	62.75

## Data Availability

Additional datasets supporting the findings of this study can be found in Supplementary Materials. Complete datasets are not publicly available but can be made available upon request, following ethics committee approval and a data transfer agreement. Please contact the author María del Pilar Navarrete-Meneses (peachnavarrete@gmail.com) to request access to the data.

## Supplementary Materials

The following supporting information can be downloaded at: https://www.mdpi.com/article/10.3390/ijms24076259/s1.
