# Supplementary material for: Exposure to Insecticides Modifies Gene Expression and DNA Methylation in Hematopoietic Tissues In Vitro
Source: Int J Mol Sci. 2023 Mar 26;24(7):6259. doi: 10.3390/ijms24076259 (PMC10094043; doi:10.3390/ijms24076259)
Supplement: Supplementary file 1 [file ijms-24-06259-s001.zip › ijms-2219679-supplementary.pdf]

Supplementary Table S1. Summary of results of over-representation test of genes with altered expression in BMMCs exposed to malathion.

**Over-representation test of UNDEREXPRESSED genes**			
		**Genes**	***p-value***
Molecular Function	Potassium cannel activity	*KCNK17, KCNA1, KCNA10, SGK2, KCNA2, LRRC52, KCNJ15, KCNMA1, KCNS3, KCNU1, WNK3, KCNG1, KCNJ6, KCNQ1, KCNB2, KCNJ8,*	3.23E-04
Protein Class	RNA binding protein	*RAVER2, SAGE1, MOV10L1, RBMXL2, SERP2, RBMY1B, MEIS1, POLR2F, LHX3, WDR33, LHX9, NOM1*	1.14E-04
**Over-representation test of OVEREXPRESSED genes**			
			p-value
Biological Process	Leukocyte chemotaxis	*FIGF, CXCL13, IL1A, CCL25, CXCL1, CCL8, CCL13, CCL7, CXCL11, CCL17, CCL3L3, CCL18, CCL22, CCL19, CXCL8, CXCL3, XCL1, CXCL9, IL36G, CCL2, CCL20, CCL1, CCL14, CXCL5, CXCL10, CSCL16, VEGFA, IL1B, CXCL2, CCL3*	2.51E-14
	Tumor necrosis factor response	*CCL25,CCL8,CCL13,CCL7,CCL17,CCL3L3,CCL18,CCL22,CCL19,XCL1,CCL2,CCL20,CCL1,CCL14,CCL3*	3.76E-03
	ERK1/ERK2 cascade positive regulation	*PDGFA, CCL25, CCL8, CCL13, CCL7 ,CCL17, CCL3L3, CCL18, CCL22, CCL19, PDGFB, XCL1, CCL2, CCL20, CCL1, CCL14, CCL3*	7.25E-08
	MAPK cascade positive regulation	*FGFR3, PDGFA, CCL25, CCL8, CCL13, CCL7, IL11,CCL17,CCL3L3,CCL18,CCL22,CCL19,PDGFB,XCL1,CCL2,CCL20,CCL1,CCL14,CCL3*	3.27E-08
	Epidermal growth factor receptor	*RHBDF2,EREG, CBLC, AREG, HBEGF, ERRFI1*	1.48E-03
	Cytokine mediated signaling	*CXCL13,TNSFF14, IL1A, TNF,CCL25, CXCL1, TNFSF15,CCL8,CCL13,CCL7,IRAK2,CXCL11,CCL17,CCL3L3,CCL18,IFITM1,CCL22,CCL19,CXCL8,CXCL3,XCL1,CXCL9,IL36G, OTUD4, CCL2, CCL20, CCL1,CCL14,CXCL5, CXCL10, LTA, SH2B2, IL1B, CXCL2, CCL3*	1.11E-12
	Toxic substance response	*PPIF, MT1A, MT1E, MT1M, MT1G, MT1F, GPX8, MT1X, MT1M, PRDX4, ADH6, SOD, CHRNA5, ACHE, MT1B, MT1HL1, MT1H, MTIL*	7.61E-04
	Cell proliferation regulation	*FIGF, PDGFA, EREG, CD80, REG1A, BTG3, IL11, CCNA1, CD38, PDGFB, CD274, LIF, AREG, BCL6B, HBEGF, VEGFA, INCA1*	1.69E-03
	MAPK cascade	*HEPACAM, IL1A, RAPGEF5, MCF2, FGFR3, FGF21, SLAMF7, CCL25, SLAMF1, RIT1, INHBA, CCL8, RASGEF1B, CCL13, CCL7, IL11, CCL17, CCL3L3, CCL18, CCL22, CCL19, FGF11, RAPGEF3, XCL1, IL36G, SPAG9, CCL2, CCL20, CCL1, CCL14*	3.24E-04
	Cell adhesion	*PARVB, ZAN, CNTN2, L1CAM, DSP, ICAM1, HEPACAM, TNS1, PCDHB11, LRRC7, ITGB8*	4.82E-04
Signaling Pathways	Interleukin signaling	*IL1A, CDKN1A, STAT1, IL3RA, IL15RA, IL2RA, AKT3, STAT4, IL11, IL4, ELK1, CXCL8, IL6, IL9, MAPK6*	7.61E-04
	CCCKR signaling	*RYR2, PDE1A, SERPINB2, PRKD1, CREM, CXCL1, SRC, REG1A, MMP7, BIRC3, ELK1, CD38, NR4A1, CXCL8, IER3, SNAI1, PTGS2, HBEGF, MMP3, CXCL2*	4.78E-05
	Chemokine and cytokine signaling-mediated inflammation	*SOCS6, PLCE1, STAT1, NFKB1, NFATC1, CCL8, AKT3, CCL13, CCL7, CCL3L3, CCL18, CCL22, CXCL8, GNAO1, GNAI1, PLCB4, XCL1, IL6, PTGS2, CCL20, REL, CCRL2, GNG4, IFNG, CXCL10, LTA, SOCS6, MYO3A, RGS1*	7.19E-05
Molecular Function	Cytokine activity	*CXCL13, TNSF14, IL1A, TNF, CCL25, CXCL1, CSF1, TNSF15, INHBA, OSM, CCL8, CCL13, CCL7, IL1, NAMPT, CXCL11, CCL17, CCL3L3, CCL18, CCL22, CCL19, PSPN, CCL8, CCL3, CSF2, GDNF, XCL1, CXCL9, IL36G, LIF, CCL2, CCL1, CCL14, CXCL5, CXCL10, LTA, CXCL16, IL1B, CXCL2, CCL3*	5.83E-13
	Growth factor binding	*SPINT3, FGFR3, IL2RA, KDR, CBLC, MET, WFIKKN2, PDGFRA, FLT1*	3.65E-04

Supplementary Table S2. Summary of results of over-representation test of genes with altered expression in PBMCs exposed to malathion

**Over-representation test of UNDEREXPRESSED genes**			
			p-value
Molecular Function	Nucleic binding	*PNLDC1, ATOH1, MARVELD2, BICC1, RBFOX1, APOBEC3A, TFCP2L1, EN1, MCMDC2, HIST1H4G, DLX1, ESR2, ZNF711, MSH4, HNF4G, DEC1, RBMY1B, TAF1B, SPRM3, RFX4, NFATC4, GATA4, TFAP2D, TCF7L2, NR1I3, TAL1, ZFP42*	9.59E-05
**Over-representation test of OVEREXPRESSED genes**			
			p-value
Biological Process	Glutamatergic synapse transmission regulation, synapse signaling	*NLGN4X, CACNB4, GABRA3, CDHR3, STX19, SYN2, NPS, HTR3C, GRM2, GABRQ, SHISA9, GRIK2, CACNB2, SCN9A, GABRG3, DLG2, SHANK2, GRM7, NLGN3, GRM3, GLRA4*	3.04E-04
	Cell-cell signaling	*FGF2, STX19, TCF7L1, SYN2, FZD10, NPS, HTR3C, GRM2, GABRQ, SHISA9, FZD7, NLGN4X, CACNB4, DACT2, GABRA3, AMER3, CDHR3, WNT1, ZNRF3, DLG2, SHANK2, DKK2, NID1, GRIK2, FGF17, CACNB2, SCN9A, GABRG3, WNT11, GRIK2*	3.61E-04

Supplementary Table S3. Summary results of over-representation test of genes with altered expression in BMMCs exposed to permethrin

	**Over-representation test of UNDEREXPRESSED genes**		
			p-value
Biological Process	Leukocyte chemotaxis	*CCL11, IL1A, CXCL1, CXCL6, CCL3L3, CXCL8, CXCL3, CCL21, IL36RN, CCL20, IL1B, CXCL2, CCL3*	7.17E-06
	Cytokine mediated signaling pathway	*CCL11, IFNA21, IL1A, TNF, CXCL1, TNFSF15, CXCL6, CCL3L3, CXCL8, CXCL3, IFNA7, CCL21, IL36RN, CCL20, IL1B, CXCL2, CCL3*	7.99E-06
	Cytokine response	*CCL11, IFNA21, IL1A, TNF, CXCL1, TNFSF15, CXCL6, CCL3L3, CXCL8, CXCL3, IFNA7, CCL21, IL36RN, CCL20, JUN, IL1B, CXCL2, CCL3*	1.64E-05
Molecular Function	Cytokine activity	*CCL11, IFNA21, IL1A, TNF, CXCL1, TNFSF15, OSM, IL31, TGFB2, NAMPT, CXCL6, A2ML1, CCL3L3, CXCL8, CXCL3, IFNA7, CCL21, IL36RN, CCL20, IL1B, CXCL2, CCL3*	2.11E-06
	Enzyme binding	*AKAP5, CARD8, SH3RF2, PRDM12, RIMS2*	1.87E-06
Protein class	Chemokine	*CCL11, CXCL1, CXCL6, CCL3L3, CXCL8, CXCL3, CCL21, CCL20, CXCL2, CCL3*	1.84E-04
	Cytokine	*CCL11, TNF, CXCL1, TNFSF15, OSM, NAMPT, CXCL6, A2ML1, CCL3L3, CXCL8, CXCL3, LEPR, CCL21, CCL20, CXCL2, CCL3D*	1.51E-04
	Ion channel	*FXYD3, TRPM1, SCN2A, TRPM3, SCN1A, TRPM8, ATP2B3, HTR3C, CHRNA9, PKD1L3, ATP2B2, CHRNA3, SCN3B, CLCA4, SCN9A, SCNBA, ATP4A, TRPC4, SCN7A, TRPV3, ATP2B2, HTR3B*	2.14E-02
**Over-representation test of OVEREXPRESSED genes**			
			p-value
Signaling pathways	Ionotropic glutamate receptor complex	*GRIN2D, GRM2, GRIN1, GRIK2, SHAK2*	4.06E-05
Molecular Function	Transmembrane signaling receptor	*OR52K1, CCL13, GRM2, OR51B5, OR1I1, OR5I1, GPR112, OPRD1, PTCH1, CGB2, TAS2R8, PREX2, OR1L3, GNG12, AVPR2, OR56A4, IL22RA2*	4.09E-06

Supplementary Table S4. Summary of results of over-representation test of genes with altered expression in PBMCs exposed to permethrin

	**Over-representation test of UNDEREXPRESSED genes**		
			p-value
Signaling pathways	Cadherin signaling	*PCDH9, PCDH15, PCDH12, WNT2, CHD19, PCDHA6, WNT4, PCDH11Y, TCF7L2, CDH19, PCDHGA5, WNT7A, PCDH7, PCDHA13, ACTG2*	1.50E-04
	Alzheimer-presenilin pathway	*LDLRAD1, ACTG2, MMP12, WNT2, WNT2, WNT4, LRP3, TCF7L2, RBPJL, WNT7A, MMP2, MMP13*	7.59E-05
Cell component	Cation channel complex	*SCN1A, KCNC2, KCNS2, GRIK2, SCN8A, KCNV1, SCN7A, GRIA4, KCNB2*	8.44E-04
	**Over-representation test of OVEREXPRESSED genes**		
			p-value
Signaling pathways	Vasopressin synthesis	*NEU2*	2.49E-05
Molecular function	Tyrosin kinase activity receptor	*EPHA7, EPHA10, RET, EPHA3, KIT, ROS1, EPHA6, PDGFRA, ALK*	1.66E-04
	RNA binding	*PABP5, RALYL, ZGRF1*	1.44E-04
Cell component	Ion channel complex	*SCN5A, GRIA3, KCNG3, KCNE4*	6.33E-04
	Nucleus	*KCNG3, KCNB1, SMDT1, KCND3, GRIA1, KCNA5, SCN9A*	1.76E-05

Supplementary Table S5 Genes with highest and lowest expression in BMMCs exposed to malathion

GeneSymbol	FC abs	Expression	GeneSymbol	Expression	FC abs
KCNMA1	11.37	down	*IL36G*	up	170.44
ADAMTS20	10.80	down	*EDN1*	up	73.34
EDNRB	9.58	down	*CYP3A5*	up	67.78
LRGUK	9.22	down	*IL6*	up	67.62
ACOT6	8.28	down	*CCL1*	up	61.38
BTNL3	7.95	down	*MMP1*	up	51.83
SFTPB	7.73	down	*MMP12*	up	51.19
KCNMA1	7.67	down	*MMP3*	up	49.73
ATP2B3	7.55	down	*CCL20*	up	47.93
NFIB	7.24	down	*IL23A*	up	39.09
OR6C2	7.21	down	*MMP10*	up	35.05
LOC100129027	7.14	down	*TFPI2*	up	33.95
MACROD2	6.99	down	*TNFSF15*	up	26.55
DTNA	6.92	down	*NEURL3*	up	24.62
MAPT-IT1	6.75	down	*CCL3*	up	24.59
LOC100128164	6.73	down	*OR11H12*	up	23.84
RPH3A	6.64	down	*ANGPTL4*	up	22.94
XKR4	6.63	down	*F3*	up	22.38
lnc-CDYL2-6	6.57	down	*C11orf96*	up	22.24
FLJ45079	6.56	down	*CXCL11*	up	22.12
SSPO	6.49	down	*TMEM158*	up	21.49
GPR179	6.45	down	*IL1A*	up	21.36
RSPO2	6.45	down	*CSF3*	up	21.06
SPANXN3	6.39	down	*HILPDA*	up	21.00
C12orf40	6.37	down	*HAS1*	up	20.72
WFDC6	6.33	down	*C15orf48*	up	19.63
TMEM72-AS1	6.25	down	*MYO1B*	up	19.24
NPIPB3	6.17	down	*ITGB8*	up	18.86
lnc-C10orf71-1	6.15	down	*TNIP3*	up	18.55
OPN5	6.15	down	*HES4*	up	18.34
ROBO4	6.07	down	*IL1B*	up	17.86
SNAR-C3	6.03	down	*PI3*	up	17.61
LINC00320	6.02	down	*MET*	up	17.58
LINC01255	5.99	down	*lnc-STEAP1B-1*	up	17.07

OR9G4	5.98	down	*CXCL8*	up	17.05
OR4F29	5.96	down	*UBD*	up	17.05
TTC9	5.92	down	*IDO1*	up	16.75
KCNMA1	5.83	down	*FAM124A*	up	16.63
LOC100131023	5.80	down	*CCL7*	up	16.61
PDE6C	5.79	down	*CCL4L2*	up	16.11
PROZ	5.78	down	*CYP27B1*	up	16.00
FGF22	5.71	down	*GBP5*	up	15.94
NNT	5.68	down	*INHBA*	up	15.67
PF4V1	5.67	down	*KRT14*	up	15.15
FLJ46120	5.63	down	*IRAK2*	up	15.11
VLDLR-AS1	5.63	down	*KRT17*	up	15.07
FAM71D	5.62	down	*CD69*	up	15.04
AMOT	5.61	down	*MT2A*	up	14.57
CEACAM7	5.58	down	*NR4A1*	up	14.51
OR4F21	5.54	down	*CYP3A7*	up	13.92
ODF2L	5.50	down	*IL11*	up	13.91
LINC01551	5.48	down	*SLCO4A1*	up	13.77
TTTY3	5.42	down	*PLIN2*	up	13.60
HOXB-AS3	5.42	down	*DKK2*	up	13.55
OR4D1	5.41	down	*TNFAIP6*	up	13.35
SORCS3-AS1	5.41	down	*RRAD*	up	13.19
TUBB8	5.38	down	*SERPINB2*	up	13.16
lnc-IL4R-2	5.33	down	*RSAD2*	up	12.99
FGG	5.30	down	*PLS3*	up	12.63
ARMC3	5.29	down	*CD274*	up	12.47
SPINK13	5.29	down	*OCSTAMP*	up	12.31
TPTE2P3	5.29	down	*CCL3L3*	up	12.20
OR2AT4	5.29	down	*CXCL5*	up	12.02
LOC646736	5.27	down	*HBEGF*	up	11.83
STK36	5.25	down	*IFI44L*	up	11.65
PROX2	5.24	down	*NEU4*	up	11.63
LOC100291323	5.22	down	*MT1B*	up	11.23
lnc-C3orf37-1	5.16	down	*CSF2*	up	11.04
TRIM48	5.15	down	*CH25H*	up	10.84
NDUFS7	5.15	down	*TNF*	up	10.82
CNTFR-AS1	5.14	down	*MT1M*	up	10.76
LOC541467	5.14	down	*MT1E*	up	10.60
LOC100130452	5.12	down	*DCN*	up	10.55
DOC2B	5.09	down	*EBI3*	up	10.49

OMG	5.08	down	*FGFBP1*	up	10.47
LOC219690	5.07	down	*NTN1*	up	10.27
SNTG1	5.06	down	*GBP3*	up	10.26
LINC00602	5.06	down	*GBP1*	up	9.91
DDR1	5.05	down	*CFB*	up	9.79
DIO1	5.04	down	*MFSD2A*	up	9.70
LOC151121	5.02	down	*IL13*	up	9.63
PITX1	5.00	down	*MT1L*	up	9.27
PCDHB12	4.99	down	*ADM*	up	9.03
C8orf34	4.99	down	*NR4A3*	up	8.97
B9D1	4.97	down	*SGPP2*	up	8.87
NEK10	4.97	down	*SGPP2*	up	8.78
TXNRD3NB	4.96	down	*GOT1L1*	up	8.77
CRISP3	4.95	down	*SCG5*	up	8.69
LOC644852	4.94	down	*LIF*	up	8.68
ADAMTS12	4.94	down	*AQP9*	up	8.65
LINGO1	4.92	down	*USP27X*	up	8.59
TRPC4	4.92	down	*C17orf96*	up	8.53
ADAM32	4.90	down	*CCM2L*	up	8.53
C3orf80	4.90	down	*DUSP5*	up	8.52
LOC1720	4.88	down	*SSTR2*	up	8.50
PCDH15	4.88	down	*CCL19*	up	8.43
PLP1	4.83	down	*IL24*	up	8.38
CAPN8	4.83	down	*LOC388780*	up	8.33
HOXA-AS2	4.83	down	*ST8SIA4*	up	8.23
IFNA2	4.81	down	*PLAUR*	up	8.18

Supplementary Table S6 Genes with highest and lowest expression in PBMCs exposed to malathion

GeneSymbol	FC abs	Expression	GeneSymbol	Fc abs	Expression
MUC12	7.21	down	*ST6GAL2*	10.95	up
RBP3	6.64	down	*RAPGEF5*	6.69	up
TNFAIP8L3	6.58	down	*PLCB4*	6.35	up
LINC00307	6.13	down	*EFCAB9*	6.29	up
PTCRA	6.04	down	*GALNT16*	5.67	up
LOC440117	5.73	down	*LEMD1*	5.61	up
SPATA17	5.59	down	*ANKRD55*	5.24	up
AKR1E2	5.55	down	*PLIN1*	5.21	up
LRRC37A6P	5.24	down	*CECR7*	5.04	up
LRP3	5.12	down	*BSND*	5.00	up
EN1	5.05	down	*LINC01183*	4.98	up
KIF6	4.97	down	*DLG2*	4.92	up
ANGPTL2	4.93	down	*LOC728339*	4.87	up
STRA6	4.88	down	*TDRD1*	4.81	up
CLDN22	4.81	down	*ERBB4*	4.75	up
LOC646034	4.81	down	*TDRD6*	4.72	up
C12orf54	4.64	down	*LCORL*	4.70	up
AMOT	4.58	down	*GP5*	4.62	up
SCN9A	4.57	down	*DSP*	4.61	up
lnc-CD99L2-1	4.56	down	*SHISA9*	4.61	up
ZNF713	4.55	down	*LINC00515*	4.52	up
PLEKHD1	4.52	down	*LINC01152*	4.51	up
TNFSF15	4.49	down	*C8orf22*	4.51	up
ANK1	4.47	down	*SHISA3*	4.46	up
LINC01255	4.45	down	*lnc-ZNF717-1*	4.45	up
CPNE9	4.44	down	*DDC*	4.40	up
CABP7	4.42	down	*RNF170*	4.37	up
CMA1	4.41	down	*ACBD5*	4.35	up
EDNRB	4.41	down	*ANKRD20A2*	4.31	up
RAB3C	4.40	down	*TCF7L1*	4.31	up
PPBPP2	4.39	down	*RELN*	4.30	up
PALMD	4.37	down	*ENKUR*	4.28	up
CEBPA-AS1	4.35	down	*LOC100288619*	4.28	up
TPD52L3	4.34	down	*CACNA1C*	4.25	up
WBP2NL	4.34	down	*IRX2*	4.23	up
C9orf117	4.34	down	*ZFPM2*	4.20	up

S100A1	4.33	down	*LIN28B*	4.18	up
LOC285857	4.32	down	*FILIP1L*	4.17	up
TIFAB	4.25	down	*GMNC*	4.16	up
RBP7	4.24	down	*ST7-AS2*	4.15	up
TMEM241	4.23	down	*EPHA10*	4.15	up
LOC643711	4.22	down	*LINC01433*	4.13	up
NPIPB3	4.20	down	*ADRA2A*	4.12	up
ARNT2	4.19	down	*CNTN4*	4.08	up
TRPC4	4.19	down	*H19*	4.02	up
HOXB-AS3	4.16	down	*FAM27B*	4.02	up
USP6	4.14	down	*MYBPHL*	4.02	up
LOC102724484	4.14	down	*ZSCAN23*	4.01	up
LYPD1	4.09	down	*ZBED3-AS1*	4.00	up
CES1P2	4.05	down	*RNF183*	4.00	up
KIAA1598	4.04	down	*GNAI1*	3.97	up
WIPF3	4.03	down	*LMBR1*	3.97	up
TMEM114	4.01	down	*RS1*	3.96	up
FAM229B	4.01	down	*LOC441666*	3.95	up
LOC100128198	4.00	down	*ADAMTS6*	3.93	up
C1QL2	3.99	down	*LINC00163*	3.93	up
DSPP	3.99	down	*DISC1*	3.93	up
LELP1	3.97	down	*CR1*	3.93	up
ZFP42	3.97	down	*SPDYA*	3.93	up
MCHR2-AS1	3.96	down	*GRK1*	3.93	up
KAZN	3.95	down	*FAM87B*	3.90	up
SLC14A1	3.94	down	*LZTS1*	3.90	up
TCHHL1	3.94	down	*DEFB108B*	3.90	up
RIMBP2	3.93	down	*KRTAP13-3*	3.89	up
LOC389332	3.93	down	*LINC00305*	3.89	up
TDRP	3.91	down	*UMODL1*	3.86	up
SST	3.89	down	*ANKRD62*	3.81	up
PDIA2	3.89	down	*STH*	3.81	up
XLOC_l2_009136	3.89	down	*USP2*	3.79	up
FAM230B	3.88	down	*MYZAP*	3.77	up
ODAM	3.88	down	*CLDN12*	3.77	up
AQP12A	3.87	down	*OMG*	3.75	up
lnc-IRF4-1	3.86	down	*CCDC126*	3.73	up
FAM227B	3.84	down	*FLT4*	3.73	up
ZSCAN5B	3.81	down	*GAS2*	3.71	up
CYMP	3.80	down	*LOC100133920*	3.70	up

SLC4A5	3.80	down	*XLOC_l2_005020*	3.69	up
CPZ	3.78	down	*LOC101930072*	3.67	up
KCNQ1DN	3.77	down	*LOC400756*	3.66	up
CROCCP3	3.75	down	*TRAM1L1*	3.66	up
GATA4	3.74	down	*TMEM65*	3.65	up
OR4F21	3.73	down	*CCSER1*	3.64	up
C10orf53	3.73	down	*PEG10*	3.61	up
LOC101448202	3.70	down	*OR2W3*	3.61	up
LOC100128164	3.68	down	*CARTPT*	3.59	up
SRP14-AS1	3.67	down	*HSF2BP*	3.59	up
SEC14L6	3.67	down	*LOC100128607*	3.59	up
LOC100131581	3.66	down	*FAM90A27P*	3.59	up
RFT1	3.65	down	*ASIC5*	3.58	up
APLF	3.65	down	*CDK15*	3.58	up
LOC100131023	3.62	down	*HCG4B*	3.57	up
MBP	3.62	down	*MGC24103*	3.57	up
PLVAP	3.62	down	*CASC11*	3.57	up
HTRA4	3.61	down	*RHOXF1*	3.57	up
SPINK4	3.61	down	*TRPC5OS*	3.57	up
lnc-ELF2-2	3.60	down	*MARCH3*	3.56	up
MEIS1	3.60	down	*PCDHGB1*	3.55	up
MAATS1	3.59	down	*KRTAP27-1*	3.53	up
HNMT	3.59	down	*KCNH5*	3.53	up
lnc-NPHS2-1	3.59	down	*FAM150B*	3.52	up

Supplementary Table S7 Genes with highest and lowest expression in BMMCs exposed to permethrin

GeneSymbol	FC abs	Exrpession	GeneSymbol	FC abs	Expression
ABCC9	16.41	down	*ST6GAL2*	7.30	up
LOC440117	8.02	down	*PPAPDC1A*	6.84	up
LOC100131599	7.43	down	*RAB43*	6.77	up
TPTE2P3	7.42	down	*TSPAN15*	6.68	up
SSTR2	7.23	down	*PTPRF*	6.55	up
PLP1	7.19	down	*LOC157273*	6.17	up
SFTPB	7.05	down	*OOEP*	5.76	up
LINC00700	7.05	down	*CROT*	5.76	up
LINC01551	7.01	down	*L2HGDH*	5.74	up
LINC00307	6.93	down	*CRYM-AS1*	5.64	up
PWRN1	6.90	down	*DCN*	5.57	up
NPIPB3	6.84	down	*FAM172BP*	5.51	up
EDNRB	6.82	down	*FANK1*	5.50	up
lnc-CDYL2-6	6.67	down	*LOC100131581*	5.47	up
CSN3	6.65	down	*TRIQK*	5.40	up
TTTY3	6.61	down	*LOC100131289*	5.38	up
SLC7A3	6.49	down	*MBD2*	5.32	up
COL6A4P2	6.47	down	*HS3ST3B1*	5.24	up
KIAA1598	6.34	down	*ZNF385D*	5.21	up
IL1A	6.23	down	*JAM2*	5.18	up
ARPIN	6.17	down	*ZNF645*	5.18	up
ATP5L2	6.13	down	*BRSK1*	5.12	up
HOXA-AS2	6.06	down	*YIF1B*	5.01	up
LOC401410	6.05	down	*CHST1*	4.83	up
XAGE2	6.01	down	*LINC00900*	4.83	up
GREM2	5.99	down	*IFIT1*	4.81	up
ADAM30	5.88	down	*GALNT16*	4.79	up
ANKRD31	5.87	down	*CD300LG*	4.78	up
FOLH1B	5.87	down	*LOC729860*	4.77	up
PDGFD	5.78	down	*GBA3*	4.76	up
DCHS2	5.74	down	*PRR27*	4.75	up
C10orf53	5.73	down	*LOC727799*	4.74	up
LOC729159	5.69	down	*TMEM72*	4.74	up
ITGB6	5.69	down	*PGC*	4.69	up
KRT40	5.66	down	*C6orf223*	4.67	up
DTHD1	5.65	down	*NFASC*	4.60	up

LOC285889	5.65	down	*LOC101930611*	4.60	up
PCDH15	5.64	down	*LOC403312*	4.56	up
PROZ	5.62	down	*IQCF1*	4.48	up
BSPH1	5.59	down	*SLC18A1*	4.47	up
LOC442028	5.59	down	*MEDAG*	4.46	up
C12orf40	5.55	down	*FBLN2*	4.43	up
OPN5	5.54	down	*UG0898H09*	4.37	up
FAM154A	5.51	down	*BDNF*	4.30	up
LOC102723778	5.49	down	*SLC9B1*	4.30	up
FABP12	5.48	down	*SMIM22*	4.29	up
C1orf141	5.47	down	*STRA6*	4.25	up
MAPRE3	5.44	down	*GDF1*	4.25	up
RPL34-AS1	5.43	down	*SAMM50*	4.24	up
CNKSR2	5.42	down	*LOC100996813*	4.24	up
CLIC6	5.41	down	*SFTA2*	4.20	up
LINC00992	5.41	down	*lnc-AC005323.1.1-1*	4.19	up
TRPV3	5.38	down	*LOC100996465*	4.18	up
LPPR4	5.38	down	*SSX2IP*	4.18	up
GPX8	5.37	down	*LOC613266*	4.17	up
FAM83C	5.34	down	*KITLG*	4.17	up
lnc-PAX9-2	5.32	down	*CD72*	4.16	up
FAM19A3	5.27	down	*OR10A6*	4.16	up
LMOD1	5.26	down	*ZDHHC23*	4.15	up
LOC285043	5.25	down	*TDRD6*	4.15	up
LINC01255	5.22	down	*IDO2*	4.14	up
LINC00207	5.22	down	*ABHD16B*	4.14	up
TRPM8	5.20	down	*AOX1*	4.13	up
LOC100129072	5.21	down	*PABPC5*	4.13	up
HMCN2	5.19	down	*LOC729451*	4.12	up
RBMXL2	5.16	down	*FAM150B*	4.12	up
LOC100131023	5.14	down	*ACBD5*	4.11	up
LOC100131497	5.14	down	*WFDC11*	4.10	up
lnc-SLC19A3-1	5.13	down	*NLRP4*	4.10	up
FGFR2	5.12	down	*LOC100129027*	4.10	up
ATP10B	5.08	down	*FIGF*	4.09	up
HS6ST3	5.04	down	*PANX2*	4.08	up
GATA4	5.04	down	*ACTL7B*	4.03	up
USP29	5.03	down	*FGD2*	4.03	up
RD3	5.03	down	*FAM230A*	4.02	up
HSD52	5.02	down	*C1QTNF1*	4.02	up

NCKAP5	5.02	down	*OR2J2*	4.01	up
GPRIN1	5.01	down	*GRM2*	4.00	up
SCN9A	5.01	down	*CGB2*	4.00	up
LOC100268168	5.00	down	*IQCC*	3.99	up
lnc-CCDC148-1	4.98	down	*PRR27*	3.99	up
AMOT	4.98	down	*PIGR*	3.97	up
MZF1-AS1	4.96	down	*KLHDC8A*	3.95	up
AJAP1	4.95	down	*TAS2R4*	3.95	up
lnc-IL4R-2	4.95	down	*IL15RA*	3.93	up
LOC100126448	4.91	down	*SLC6A20*	3.93	up
TPTE2	4.90	down	*PARD3*	3.91	up
PLA2G10	4.90	down	*KLB*	3.90	up
EFCAB10	4.89	down	*GJA9*	3.89	up
CA3	4.87	down	*TRAM1L1*	3.88	up
TBX15	4.86	down	*PFKFB1*	3.87	up
ZNF569	4.85	down	*EGFEM1P*	3.87	up
LINC01102	4.82	down	*LOC728752*	3.86	up
THSD4	4.81	down	*LOC388820*	3.85	up
RGS5	4.80	down	*LOC100134368*	3.85	up
lnc-LBH-1	4.75	down	*LINC00910*	3.82	up
LOC151121	4.75	down	*RAB26*	3.81	up
KIAA1549L	4.75	down	*C1orf86*	3.79	up
PCDH9	4.74	down	*CDKL4*	3.78	up
THBS2	4.74	down	*DBF4B*	3.78	up

Supplementary Table S8 Genes with highest and lowest expression in PBMCs exposed to permethrin

GeneSymbol	FC abs	Expresssion	GeneSymbol	FC abs	Expression
lnc-FAM189A2-1	6.32	down	*BLOC1S5*	17.43	up
PWRN1	5.98	down	*ST6GAL2*	16.07	up
LOC51145	5.77	down	*NPSR1*	7.57	up
STRA6	5.70	down	*POU5F2*	7.06	up
TUBB8	5.64	down	*SPRR1A*	6.04	up
LOC440386	5.37	down	*ZNF385D*	5.96	up
LOC401220	5.33	down	*UPB1*	5.93	up
RSPH6A	5.24	down	*RBMX2*	5.84	up
lnc-TMEM30B-2	5.21	down	*DISC1*	5.68	up
FLJ36000	5.09	down	*SCART1*	5.52	up
CPXM2	5.03	down	*SYN2*	5.47	up
EPX	5.02	down	*LINC00696*	5.17	up
C17orf67	4.96	down	*PCDHA8*	5.10	up
COL8A1	4.92	down	*WNT11*	5.08	up
SLC14A1	4.91	down	*RAPGEF5*	4.96	up
KAAG1	4.89	down	*PER3*	4.95	up
IL13RA2	4.80	down	*KLK8*	4.94	up
LOC541467	4.73	down	*CNKSR2*	4.88	up
MYL1	4.68	down	*LOC441666*	4.85	up
FUNDC2	4.65	down	*ST8SIA4*	4.85	up
OR1L8	4.61	down	*LINC00371*	4.83	up
EFCAB8	4.58	down	*HMGA2*	4.81	up
C10orf105	4.57	down	*ZDHHC23*	4.81	up
FBXL2	4.57	down	*RHBDL2*	4.78	up
ANKRD6	4.57	down	*TRIQK*	4.76	up
GRIP2	4.51	down	*DSP*	4.74	up
WLS	4.47	down	*ARSK*	4.64	up
MIR99AHG	4.47	down	*FOXI1*	4.60	up
LOC389332	4.45	down	*KDM5A*	4.59	up
A2ML1	4.39	down	*SULT1C2P1*	4.59	up
B3GALT2	4.38	down	*HBBP1*	4.55	up
LOC100129175	4.38	down	*ZBED3-AS1*	4.52	up
CAPN8	4.35	down	*PCDHB18P*	4.48	up
LOC100128402	4.34	down	*PID1*	4.46	up
LOC102723895	4.32	down	*PDHA2*	4.46	up
LRRC1	4.31	down	*SHISA3*	4.42	up

ARPIN	4.29	down	*TRAM1L1*	4.41	up
TMEM247	4.29	down	*TAS2R20*	4.40	up
LOC101929465	4.27	down	*RGSL1*	4.38	up
FRMPD2	4.26	down	*NBPF25P*	4.38	up
IFIT1B	4.23	down	*SFTPB*	4.35	up
SPATA17	4.18	down	*MYT1*	4.34	up
LOC389834	4.17	down	*PPP1R17*	4.33	up
ROBO1	4.16	down	*C2orf27B*	4.27	up
LOC100128164	4.16	down	*HIST2H2BF*	4.26	up
WBSCR28	4.16	down	*GAB1*	4.25	up
C3orf30	4.13	down	*ZNF43*	4.25	up
LINC01555	4.13	down	*VGLL3*	4.25	up
LOC100129775	4.13	down	*ELOVL4*	4.24	up
TPK1	4.08	down	*ANKRD20A12P*	4.22	up
IFNA6	4.06	down	*PLG*	4.19	up
MMP13	4.05	down	*TNFSF11*	4.17	up
KLHDC8A	4.04	down	*FAP*	4.16	up
LOC730668	4.02	down	*lnc-FGFR1OP-2*	4.14	up
lnc-DIO2-5	4.01	down	*PABPC5*	4.12	up
PRUNE2	4.01	down	*EPHA10*	4.09	up
LOC102724030	4.00	down	*GRIA1*	4.08	up
KIAA1024L	4.00	down	*FABP9*	4.07	up
LOC102724921	3.99	down	*EPHA6*	4.04	up
LEKR1	3.99	down	*SPACA5*	4.04	up
SLC9C1	3.98	down	*KRTAP27-1*	4.04	up
CYYR1	3.98	down	*ZNF703*	4.02	up
DIO1	3.98	down	*PPEF1*	3.99	up
CP	3.96	down	*PRR23B*	3.97	up
OR5E1P	3.95	down	*LOC340113*	3.92	up
SEMA5B	3.95	down	*BTNL3*	3.92	up
AGVR6190	3.94	down	*TBX5-AS1*	3.90	up
WBP2NL	3.92	down	*lnc-SART3-1*	3.90	up
KC6	3.91	down	*CCDC17*	3.90	up
SAMD7	3.91	down	*MROH2A*	3.89	up
ANGPTL1	3.90	down	*PRSS55*	3.89	up
SGCD	3.89	down	*GRID1*	3.88	up
SOX18	3.89	down	*LINC00620*	3.86	up
XKR4	3.88	down	*FLJ41170*	3.84	up
MXRA5	3.87	down	*LINC00518*	3.82	up
C9orf135-AS1	3.87	down	*LOC646522*	3.82	up

LINC00083	3.86	down	*CT83*	3.80	up
LOC339862	3.86	down	*LOC339975*	3.80	up
AADACL4	3.85	down	*GJB3*	3.80	up
LOC649133	3.82	down	*LCORL*	3.77	up
GJA10	3.81	down	*CEACAM22P*	3.77	up
LINC01551	3.80	down	*CLPSL1*	3.74	up
LOC100130642	3.78	down	*lnc-CYS1-3*	3.73	up
GJD2	3.78	down	*PLCH1*	3.72	up
PSAPL1	3.77	down	*IRX2*	3.70	up
SULT1C2	3.77	down	*LINC00473*	3.69	up
PART1	3.75	down	*MMP7*	3.65	up
HTR5A-AS1	3.74	down	*PUS7L*	3.64	up
PRX	3.74	down	*C9orf171*	3.64	up
LOC643441	3.73	down	*XDH*	3.64	up
SULT1C4	3.72	down	*PRRG1*	3.61	up
LOC401410	3.72	down	*LINC01097*	3.61	up
MAPK4	3.71	down	*LINC00376*	3.60	up
LINC00052	3.71	down	*HOXB5*	3.58	up
RFPL2	3.71	down	*ALS2CR11*	3.58	up
KRTAP26-1	3.68	down	*ATG10*	3.54	up
MGAM	3.68	down	*FNDC7*	3.53	up
LHX3	3.66	down	*LOC728339*	3.53	up
OR5D18	3.65	down	*FAM110C*	3.53	up
C14orf119	3.65	down	*SLC44A5*	3.52	up

Supplementary Table S9. Genes selected for methylation analysis.

**Genes with under expression in BMMCs exposed to malathion**	**Genes with over expression in BMMCs exposed to malathion**	**Genes with under expression in BMMCs exposed to permethrin**	**Genes with over expression in BMMCs exposed to permethrin**
NFIB	*F3*	*ADAM3O*	*ST6GAL2*
ADAM32	*IDO1*	*PCDH9*	*MBD2*
FGF22	*IRAK2*	*FGFR2*	*KITLG*
KCNMA1	*SLCO4A1*	*ABCC9*	*FIGF*
ADAMTS20	*DUSP5*	*ANKRD31*	*IL15RA*
AMOT	*PLAUR*	*PDGFD*	*BDNF*
TRPM6	*TFPI2*	*TRPM8*	*SLC6A20*
STK36	*IL15RA*	*GATA4*	*GDF1*
RSPO2	*MYO1B*	*SCN9A*	*KLB*
EDNRB	*INHBA*	*AJAP1*	*PANX2*
TUBB8	*HES4*	*TBX15*	*JAM2*
ADAM11	*EDN1*	*USP29*	*PARD3*
PITX1	*LIF*	*LMOD1*	*MAPK*
LINGO1	*CSF2*	*MAPRE3*	*IFI27*
LRIG3	*NR4A3*	*TBX15*	*FBLN2*
DDR1	*NR4A1*	*RBMXL2*	*TDRD6*
PROZ	*CCL19*	*FGFR2*	
	ADM	*MAPRE3*	
	ANGPTL4	*GATA4*	
	MET	*LMOD1*	
	ITGB8	*ADAM30*	
		PROZ	
		DCHS2	
		EDNRB	

*Genes were selected according to their relevance in cancer and because significant results were observed in the expression analysis.

Supplementary Table S10. Primers sequences used for qPCR expression analysis.

GEN	REF	OLIGONUCLEÓTIDO SENTIDO	OLIGONUCLEOTIDO ANTISENTIDO	SONDA
PCDH15	NM_001142771	CAGGAGGAATATGGTGAGGTG	CATTCTTTTTCTTGCCCACTCT	#73
PCDH9	NM_020403	TGGAACAAATCATTCCTAACTGC	TCTCATCACTTATTTGGAGACAGC	#48
IL6	NM_0006000	GATGAGTACAAAAGTCCTGATCCA	CTGCAGCCACTGGTTCTGT	#40
DSP	NM_004415	CTTTGCGCCAATTCAATTAAG	CCAGTCCTGAGGTGTATGAGG	#78
LYPD1	NM_144586	CGGATTGTTCTTGCTTCCAG	CCGTGCAATTCACAATGAAC	#52

Supplementary Table S11. Biological relevance and role in cancer of selected genes with altered expression in cells exposed to insecticides.

Gene	Biological relevance and Reports of the effect of insecticides	Relevance in cancer
**Genes repressed in BMMCs exposed to malathion**		
KCNMA1<br>Gene of the calcium-activated potassium channel family. Muscular tone control and neuronal excitability.	*KCNMA1* alterations detected in individuals with hypokalemia and paralysis. Individuals exposed to organophosphates, show low potassium levels. Hypokalemia could be a predictive marker of poisoning with organophosphates. Endosulfan also represses *KCNMA1* expression<br>(33,34)	
ADAM11, ADAM32<br><br>Disintigrin and metalloproteinase family of genes		*ADAM 11* is a tumor suppressor. Repressed in cancer cells, associated with invasion and metastasis [35]
PCDH12, PCDH15<br>Cell-cell adhesion function<br>Mainly nervous system	Murine models showed that organophosphates can lead to the deregulation of protocadherins [36]	Participate in proliferation and death.<br>Hypermethylation of members of the protocadherin family has been reported in pre-B ALL and other types of cancer.<br>Can act as tumor suppressors through WNT pathway [37]
EDNRB<br>Encodes endothelin	Murine models showed that chlopyrifos inhibits *EDNRB* expression in brain cells [38]	Hypermethylation reported in different types of leukemia [37]
**Genes overexpressed in BMMCs exposed to malathion**		
IL6<br>Pro-inflammatory cytokinne	Released by monocytes, macrogphages, T lymphocytes during chronic inflammation.<br>Released by HSCs in response to stressors. [41,42]	Increase of IL6 levels in patients with pre-leukemic and leukemic conditions. Component of tumor microenvironment. It Could promote the progression of disease [41].
TNF-α<br>Promotes de production of IL6		Participates in transformation, proliferation, and extramedullary infiltration of leukemogenesis.<br>It is an essential factor for tumor microenvironment which permits the leukemic cells to evade the immune response, to survive and resist the treatment [42].<br>Increased expression in pre-B ALL [43].
**Genes repressed in PBMCs exposed to malathion**		
REX1<br>Transcription factor. Marker of embryonic stem cells. Regulates proliferation of human mesenchymal cells through MAPK signaling.	*REX1* suppression leads to overexpression of proteins of MAPK pathway [49]. In PBMCs exposed to permethrin, the expression of *MAP4K3* was increased.	
MEIS1<br>Transcription factor vital in normal hematopoiesis.	Regulates the response to oxidative stress and metabolism of HSCs. The repression of *MEIS1* in LT-HSCs (Long-term hematopoietic stem cells] drives cells to a higher intake of oxygen and less glycolytic flux, generating ROS through a mechanism dependent on HIF-1α and HIF-2α [50, 51].	Essential role in leukemogenesis

EDNRB<br>Encodes endothelin	Murine models showed that chlorpyrifos inhibits *EDNRB* expression in brain cells [38]	Hypermethylation reported in different types of leukemia [37]
**Genes overexpressed in PBMCs exposed to malathion**		
GRIK2<br>Encodes kainic acid type glutamate receptor, mainly expressed in central nervous system.	B cells also express *GRIK2*, potentiating cell activation.	Increased levels of glutamate can promote the proliferation of histolytic lymphoma, and participation of glutamate receptors in signaling has been observed in other types of cancer. *GRIK2* has been found hypermethylated in LLA and is considered a tumor suppressor gene [52,53].
WNT1, WT11		WNT is an essential pathway in leukemia and lymphoma [54]. Deregulation of WNT pathway genes has been detected in LLA. *WNT11* promotes the migration of cancer cells, and its expression seems influenced by ERRα and β catenin [55].
**Genes repressed in BMMCs exposed to permethrin**		
CXCL8<br>Chemokine		Antitumor role [56].
OSM		Inhibits myeloid leukemic cell proliferation and modulates the differentiation of various cell types [57].
TGFB2<br>Belongs to the family of TGF proteins and has an essential role in hematopoiesis regulation, because it negatively regulates proliferation and stimulates differentiation and apoptosis when it is needed.	It is suggested that neurotoxic insecticides can alter the expression of genes of the TGFB pathway; however, this has not been previously reported for pyrethroids [58].	In different hematopoietic cancers, deregulation of the TGFB pathway and tumor suppressor function has been described [59]
GPX8<br>This gene encodes a peroxidase glutathione protein (GPx). GPx proteins regulate oxidative stress, having antioxidant activity by removing free radical excess.	The low expression of *GPX8* may enable permethrin toxicity through the accumulation of ROS and lipid peroxidation. It has been reported that insecticides such as chlorpyrifos induce low expression of the *GPX8* gene [60].	
GATA4<br>Belongs to the family of transcription factors with zinc finger motifs and has a vital role in cardiac, respiratory, gastrointestinal systems and cancer.		Has been recognized as a tumor suppressor gene in different types of cancer. Particularly in pediatric leukemia, hypermethylation of the promoter has been detected, although its function in the disease's etiology has not yet been defined [61].
SCN2A, SCNN1, SCN9A, SCN3B<br>Voltage-dependent sodium channels	These results are relevant because it is known that pyrethroids act on sodium and potassium ion channels to exert their neurotoxic action [62].	
PCDH15, PCDH9<br>Protocadherins		Deregulation of these genes is a characteristic of leukemic cells [43].
**Genes overexpressed in BMMCs exposed to permethrin**		

MBD2<br>Nucleic acid binding gene.	It can activate gene expression by recruiting HAT (Histone acetyltransferases) proteins and other transcriptional factors such as TACC3; it can promote DNA demethylation [64] This could partly explain the high frequency of promoters with demethylation observed in BMMCs exposed to permethrin, concordant with an increase in gene expression.	
IFIT1<br>Type1 Interferon family, which participates in biological functions such as defense mechanisms, apoptosis, and inflammation.		It has been reported that *IFIT1* activation can occur through the JAK/STAT pathway signaling, and it is suggested that high expression of this gene could be a useful biomarker in the diagnosis of myelodysplastic syndromes, before the development of leukemia, because it is an event that precedes the transformations towards acute myeloid leukemia [65,66]. Additionally, the activation of the JAK/STAT pathway is relevant in pre-B ALL.
**Genes repressed in PBMCs exposed to permethrin**		
WNT2, WNT4, WNT7A		WNT7A gene participates in carcinogenesis and progression of diverse types of cancer, although its function has been controverted because it has shown repression in certain cases [47,67]. Moreover, in pre-B ALL *ETV6-RUNX1* positive, hypermethylation of *WNT2* has been described.
PCDH9, PCDH15, PCDH12, PCDH114, PCDHGA5<br>Protocadherins. Cell-cell adhesion proteins mainly found in the central nervous system<br>***CADH19, TCF7L2***<br>Cadherin signaling	Participate in aging	Participate in cancer. Although the role of protocadherins is variable, in some studies, hypermethylation has been detected and thus, they are considered a tumor suppressor [68]. In ALL *BCR-ABL1* positive, hypermethylation of PCDH12 has been observed [42].
PRUNE2		This gene participates in cell death. This gene has been identified in other types of cancer as a tumor suppressor, and has been determined that its activation leads to an increase in the proliferation of tumor cells [69].
MMP12, MMP2, MMP13<br>Metalloproteins.	Participate in Alzheimer's development. Pyrethroids are also associated with the development of this disease. The potential of permethrin to repress genes coding metalloproteinases could be involved in the mechanism by which this pyrethroid induces Alzheimer's and other neurodegenerative diseases [70]	
**Genes overexpressed in PBMCs exposed to permethrin.**		
NEU2<br>Encodes sialidase protein.	Overexpression of *NEU2* impedes cell growth, blocking different cell cycle checkpoints and affecting all cell cycle phases. NEU2 can also inhibit the PI3K pathway and activate apoptosis through Fas [73].	The expression of *NEU2* is almost undetectable in most tissues. Involvement of this gene in prostate cancer has been reported, in which it has been found repressed.
ROS1<br>Tyrosine kinase receptor family		Suffers abnormal activation on CD34+ cells in most cases diagnosed as chronic myelomonocytic leukemia, leading to the

		activation of the Erk/Akt pathway through the Grb2/SOS complexes [74].
KIT<br>Tyrosine kinase receptor family		Normally expressed in hematopoietic stem and progenitor cells of bone marrow, and it is almost not expressed in differentiated cells of peripheral blood. However, the overexpression of *KIT* has been reported in many tumors. In leukemic cells, the expression of *KIT* promotes malignant phenotype. *KIT* plays an essential role in diverse signaling pathways, including PI3K, Ras-Erk, and JAK/STAT, which lead to cancer cell proliferation activation, survival, growth progression, and migration [75].
ALK<br>Tyrosine kinase receptor family		Overexpressed in diverse neoplasia, including blood cancer. *ALK* signaling activates the PI3K/AKT pathways, MAPK/ERK, and STAT*3*, promoting cell growth, survival, and metastasis of the cancer cell [76].
